# *E2F2/5/8* Serve as Potential Prognostic Biomarkers and Targets for Human Ovarian Cancer

**DOI:** 10.3389/fonc.2019.00161

**Published:** 2019-03-22

**Authors:** Quan Zhou, Fan Zhang, Ze He, Man-Zhen Zuo

**Affiliations:** Department of Gynecology and Obstetrics, The People's Hospital of China Three Gorges University/The First People's Hospital of Yichang, Yichang, China

**Keywords:** E2Fs, ovarian cancer, database mining, prognostic value, bioinformatics analysis

## Abstract

*E2Fs* are a family of pivotal transcription factors. Accumulative evidence indicates that aberrant expression or activation of *E2Fs* is a common phenomenon in malignances, and significant associations have been noted between *E2Fs* and tumorigenesis or progression in a wide range of cancers. However, the expression patterns and exact roles of each *E2F* contributing to tumorigenesis and progression of ovarian cancer (OC) have not yet been elucidated. In this study, we investigated the distinct expression and prognostic value of E2Fs in patients with OC by analyzing a series of databases, including ONCOMINE, GEPIA, cBioPortal, Metascape, and Kaplan–Meier plotter. The mRNA expression levels of *E2F1/3/5/8* were found to be significantly upregulated in patients with OC and were obviously associated with tumor stage for OC. Aberrant expression of *E2F2/5/7/8* was found to be associated with the clinical outcomes of patients with OC. These results suggest that *E2F2/5/8* might serve as potential prognostic biomarkers and targets for OC. However, future studies are required to validate our findings and promote the clinical utility of *E2Fs* in OC.

## Introduction

Ovarian cancer (OC) is a commonly diagnosed gynecological malignancy with the highest cancer-related death rate worldwide (1). According to the development trend, the lifetime incidence for ovarian malignancies is 1 in 72 (1.39%), and the lifetime risk of death from OC is 1 in 96 (1.04%) for women (2). In 2018, approximately 22,240 new cases of OC were diagnosed, and 14,070 OC-related deaths occurred in the United States (3). The high lethality rate can be attributed to the lack of effective biomarkers to detect the disease and to predict the outcome for heterogeneous biological subgroups of patients. Over 75% of patients are not diagnosed until the disease is advanced (stages III and IV), for which the 5-year overall survival (OS) rate is below 30 % (2, 4, 5). Thus, identifying reliable predictive biomarkers for early diagnosis and precise prognosis and developing novel molecular-targeted therapeutic strategies for OC are urgently required.

At present, several predictive biomarkers, which might have potential diagnostic, prognostic, or therapeutic values for OC, have been reported. Some of these markers include osteopontin, mesothelin, vascular cell adhesion molecule-1, kallikreins, *B7-H4*, human prostasin, apolipoprotein A1, interleukin*-*6/8, glutathione *S*-transferase polymorphisms, folate receptor alpha miRNA, and aldehyde dehydrogenase (6). Although some of the abovementioned biomarkers of OC have attracted considerable attention, most of them were investigated individually and not as a part of the entire oncogenesis process; related studies are still in the preliminary investigation or clinical validation stage. Further, these potential OC biomarkers do not play a significant role in improving the screening, diagnosis, prognosis, prevention, and therapy of OC (3, 4).

*E2Fs*, a group of genes that encode a family of transcription factors in higher eukaryotes, are widely expressed in many tissues and organs (7). The *E2F* family includes eight members: *E2F1* to *E2F8* (8). The members have different homology, which apparently affects their function; hence, the *E2F* family is divided into the following two subfamilies: *E2F1-3* are activators of transcription, whereas *E2F4-8* act as repressors (9). The molecular functions of *E2Fs* are cellular proliferation, differentiation, DNA repair, cell cycle regulation, and cell apoptosis (10). Increased aberrant expression or activation of *E2F*s has been reported in several human malignancies; in some studies, *E2F*s might act as promising biomarkers to predict tumor prognosis (9–11). Therefore, identifying the underling mechanisms of *E2F*-mediated oncogenes or tumor suppressors as predictive biomarkers might provide novel therapeutic strategies. Several *E2Fs* were shown to be deregulated in OC compared with that in normal tissues, and high expression levels of *E2F1, E2F2, E2F4, E2F7*, and *E2F8* were found to be significantly associated with survival rate in OC (12, 13). More importantly, *E2F1* and *E2F2* have attracted increasing attention as targeted molecular therapeutic genes for OC (13–16). However, the differences in expression levels, genetic alterations, biological functions, molecular mechanisms, and prognostic significance of the majority of E2Fs in OC have not yet been completely elucidated.

The development of microarray and RNA-sequencing technology has revolutionized RNA and DNA research, which has become a crucial component of biological and biomedical research (17, 18). In this study, we extended the knowledge related to OC based on various large databases for conducting the comprehensive analysis of the relationships between the eight E2F subtypes and the pathogenesis and progression of OC.

## Materials and Methods

### ONCOMINE Analysis

The gene expression array datasets of ONCOMINE (www.oncomine.org) are a publicly accessible, online cancer microarray database that helps facilitate research from genome-wide expression analyses. ONCOMINE was used to analyze the mRNA levels of *E2F* family members in OC (19, 20). The thresholds were restricted as follows: *P*-value = 0.001; fold-change = 1.5; and data type, mRNA. For each gene, comparison between cancer specimen and normal control dataset analysis was performed.

### GEPIA Dataset Analysis

Gene Expression Profiling Interactive Analysis (GEPIA) is an interactive web server for estimating mRNA expression data based on 9,736 tumors and 8,587 normal samples in the Cancer Genome Atlas (TCGA) and Genotype-tissue Expression dataset projects. GEPIA provides key interactive and customizable functions, including differential expression analysis, profiling plotting, correlation analysis, patient survival analysis, similar gene detection, and dimensionality reduction analysis (21).

### TCGA and CBioPortal Analysis

The cBioPortal for cancer genomics (http://www.cbioportal.org/) is affiliated with the Memorial Sloan Kettering Cancer Center and provides information regarding the integrative analysis of complex cancer genomics and clinical profiles from 105 cancer studies in the TCGA pipeline (22). The frequency of *E2F* family gene alterations (amplification, deep deletion, and missense mutations), copy number variance obtained from Genomic Identification of Significant Targets in Cancer(GISTC), and mRNA expression z-scores (RNA Seq V2 RSEM) were assessed using the cBioPortal for Cancer Genomics database and TCGA. In addition, co-expression and network analyses were performed according to the online instructions of cBioPortal (23).

### Functional Enrichment Analysis

Metascape (http://metascape.org) is a free, well-maintained, user-friendly gene-list analysis tool for gene annotation and analysis. It is an automated meta-analysis tool to understand common and unique pathways within a group of orthogonal target-discovery studies. In this study, Metascape was used to conduct pathway and process enrichment analysis of *E2F* family members and neighboring genes significantly associated with E2F alterations. For this, the Gene Ontology (GO) terms for biological process, cellular component, and molecular function categories, as well as Kyoto Encyclopedia of Genes and Genomes (KEGG) pathways, were enriched based on the Metascape online tool. Only terms with *P*-value < 0.01, minimum count of 3, and enrichment factor of >1.5 were considered as significant. The most statistically significant term within a cluster was chosen as the one representing the cluster. A subset of enriched terms was selected and rendered as a network plot to further determine the relationship among terms, where terms with similarity of >0.3 were connected by edges. Protein–protein interaction enrichment analysis was performed using the following databases: BioGrid, InWeb_IM, and OmniPath. Further, Molecular Complex Detection (MCODE) algorithm was applied to identify densely connected network components.

### The Kaplan–Meier Plotter Analysis

Kaplan–Meier plotter (www.kmplot.com) is an online database containing microarray gene expression data and survival information derived from Gene Expression Omnibus, TCGA, and the Cancer Biomedical informatics Grid, which contain gene expression data and survival information of 1,816 clinical OC patients (24). In this study, the prognostic value of the mRNA expression of *E2F* family members was evaluated using the Kaplan–Meier plotter. The OS, progression-free survival (PFS), and post-progression survival (PPS) of patients with OC were determined by dividing the patient samples into two groups based on median expression (high vs. low expression) and assessing using a Kaplan–Meier survival plot, with a hazard ratio with 95% confidence intervals and log rank *p*-value. Subgroup analyses were performed by dividing patients based on pathological and histological subtypes.

## Results

### Transcription Levels of *E2Fs* in Patients With OC

**Figure 1:**
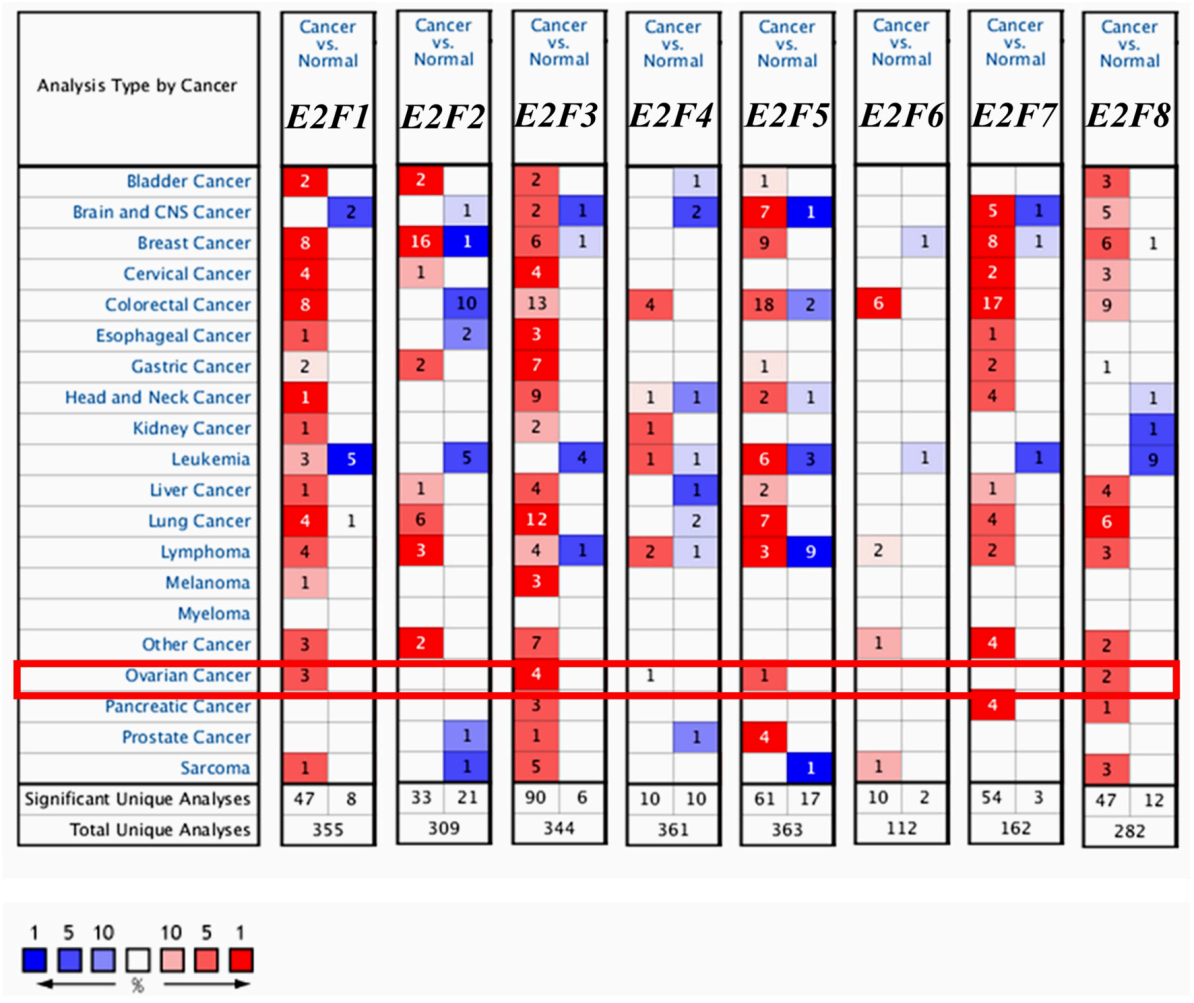
The transcription levels of *E2F* family members in different types of cancers (ONCOMINE). The graph shows the numbers of datasets with statistically significant mRNA over-expression (red) or down-regulated expression (blue) of the target gene. The threshold was designed with following parameters: *p*-value of 1E-3 and fold change of 1.5.

Eight *E2F* family members have been identified in human cancers. We compared the transcriptional levels of *E2Fs* in cancers with those in normal tissue samples by using ONCOMINE databases (Figure 1 and Table 1). ONCOMINE analysis revealed that the mRNA expression of *E2F1, E2F4, E2F5*, and *E2F8* was upregulated in patients with OC. The transcription levels of *E2F1* were significantly higher in patients with OC in three datasets (25, 26). In Yoshihara's dataset (26), *E2F1* was overexpressed in ovarian serous adenocarcinoma compared with that in the normal samples, with a fold change of 26.734 and *p–value* of 6.79E-05. In Bonome's dataset (25), *E2F1* was overexpressed in ovarian carcinoma with a fold change of 1.644 and *p–value* of 2.60E-07. In the TCGA dataset, *E2F1* was overexpressed in ovarian serous carcinoma compared with that in the normal samples, with a fold change of 1.639 and *p–value* of 1.29E-06. The transcription levels of E2F3 were significantly higher in patients with OC in four datasets (26–28). In the TCGA dataset, the fold change of mRNA expression of *E2F3* in ovarian serous carcinoma was 2.013 and *p–value* of 1.96E-11. In Welsh's dataset (27), *E2F3* was upregulated in ovarian serous carcinoma with a fold change of 2.574 and *p–value* of 3.40E-07. In Lu's dataset (28) and Yoshihara's dataset (26), *E2F3* was significantly overexpressed in ovarian serous adenocarcinoma with fold changes of 1.838 (*p–value* = 1.27E-04) and 1.833 (*p–value* = 5.66E-04), respectively. The mRNA levels of *E2F4* in ovarian carcinoma (fold change = 1.573 and *p–value* = 7.77E-04) and ovarian serous carcinoma (fold change = 2.574 and *p–value* = 1.01E-06) were significantly higher than those in the normal samples in Bonome's (25) and Welsh's datasets (27). The transcriptional levels of *E2F5* in ovarian carcinoma (fold change = 4.355 and *p–value* = 2.71E-08) were significantly different from those in the normal samples in Yoshihara's dataset (26). A similar trend was found for *E2F8* in Lu's (28) and TCGA datasets: the mRNA levels of *E2F8* in ovarian serous adenocarcinoma (fold change = 1.771 and *p–value* = 6.04E-05) and ovarian serous carcinoma (fold change = 3.136 and *p–value* = 7.97E-06) were significantly higher than those in the normal samples. In addition, no significant difference in *E2F2, E2F6*, and *E2F7* mRNA expression was found between OC and normal controls, according to ONCOMINE analysis. Although the transcription levels of E2F2 were also slightly higher than those in normal ovarian tissues with *p*-value of no more than 0.05, the cut-off of fold change was <1.5.

**Table 1:** The transcription levels of E2F family members in between different types of OC and ovarian normal tissues (ONCOMINE).

	**Types of ovarian cancer vs. norma**	**Fold change**	***t*-test**	***p-value***	**Ref**	**PMID**
E2F1	Ovarian serous carcinoma vs. normal	26.734	6.95	6.79E-05	Yoshihara ovarian	19486012
	Ovarian carcinoma vs. normal	1.644	10.596	2.60E-07	Bonome ovarian	18593951
	Ovarian serous carcinoma vs. normal	1.218	8.593	7.69E-06	Hendrix ovarian	16452189
	Ovarian serous carcinoma vs. normal	1.195	7.539	1.26E-05	Hendrix ovarian	16452189
	Ovarian serous carcinoma vs. normal	1.118	4.551	8.56E-04	Hendrix ovarian	16452189
	Ovarian serous carcinoma vs. Normal	1.639	11.563	1.29E-06	TCGA ovarian	
E2F2	Ovarian carcinoma vs. normal	1.091	3.188	3.00E-03	Bonome ovarian	18593951
E2F3	Ovarian serous carcinoma vs. normal	2.013	22.413	1.96E-11	TCGA ovarian	
	Ovarian serous carcinoma vs. normal	2.574	11.888	3.40E-07	Welsh ovarian	11158614
	Ovarian serous carcinoma vs. normal	1.838	5.143	1.27E-04	Lu ovarian	15161682
	Ovarian serous carcinoma vs. normal	1.833	3.901	5.66E-04	Yoshihara Ovarian	19486012
	Ovarian serous carcinoma vs. normal	1.176	8.187	1.04E-04	Hendrix Ovarian	16452189
	Ovarian serous carcinoma vs. normal	1.138	6.236	2.13E-04	Hendrix ovarian	16452189
	Ovarian serous carcinoma vs. normal	1.429	5.945	1.88E-04	Hendrix ovarian	16452189
E2F4	Ovarian carcinoma vs. normal	1.573	4.417	7.77E-04	Bonome ovarian	18593951
	Ovarian serous carcinoma vs. normal	1.203	9.917	1.25E-04	Hendrix ovarian	16452189
	Ovarian serous carcinoma vs. normal	1.231	11.123	6.90E-05	Hendrix ovarian	16452189
	Ovarian serous carcinoma vs. normal	1.228	8.276	8.32E-06	Hendrix Ovarian	16452189
	Ovarian serous carcinoma vs. normal	1.074	5.971	1.96E-07	Hendrix ovarian	16452189
	Ovarian serous carcinoma vs. normal	2.574	11.888	1.01E-06	Welsh ovarian	11158614
E2F5	Ovarian serous carcinoma vs. normal	4.355	8.753	2.71E-08	Yoshihara ovarian	19486012
	Ovarian serous carcinoma vs. normal	1.431	5.429	1.11E-05	Lu ovarian	15161682
	Ovarian serous carcinoma vs. normal	1.148	5.357	6.56E-06	Hendrix ovarian	16452189
E2F6						
E2F7						
E2F8	Ovarian serous carcinoma vs. normal	1.771	5.173	6.04E-05	Lu ovarian	15161682
	Ovarian serous carcinoma vs. normal	3.136	9.63	7.97E-06	TCGA ovarian	

**Figure 2:**
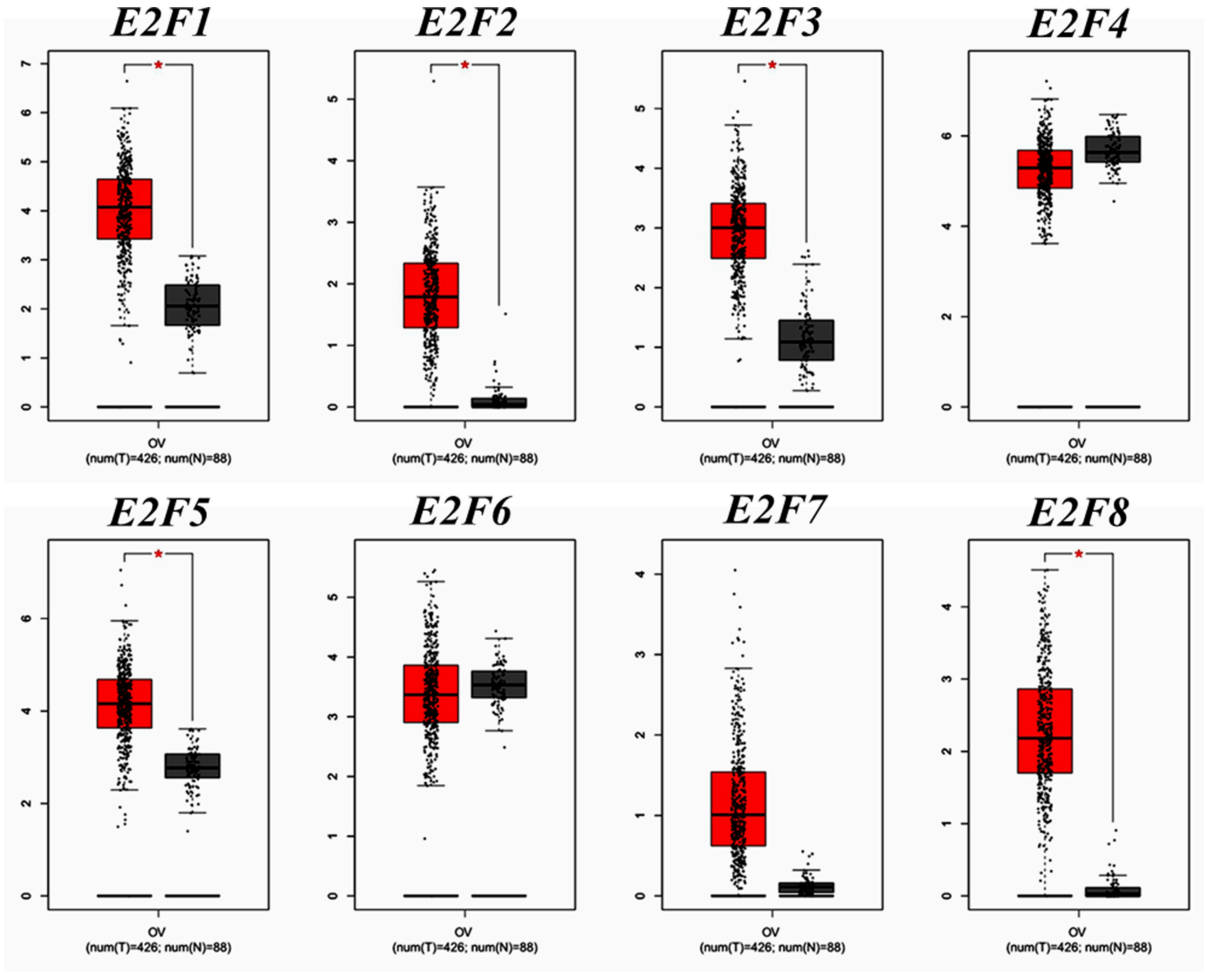
The expression of *E2F* family members in OC patients (GEPIA). Box plots derived from gene expression data for GEPIA comparing the expression of a specific *E2F* family member in OC tissue and normal tissues; the *p*-value was set at 0.05. The mRNA expression pattern of *E2F1, E2F2, E2F3, E2F4, E2F5, E2F6, E2F7*, and *E2F8* between OC and normal tissues. ^*^Indicate that the results are statistically significant.

**Figure 3:**
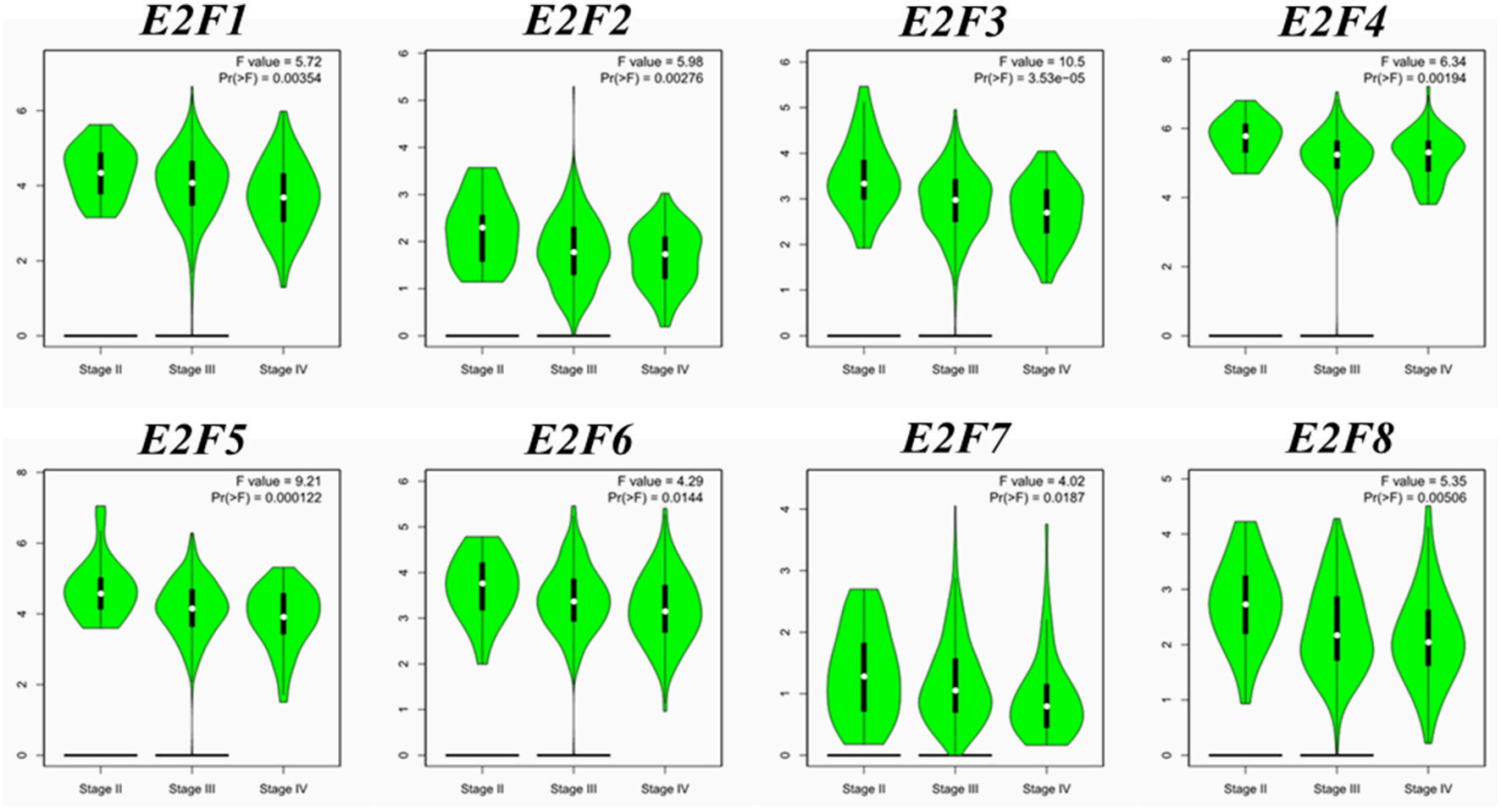
Correlation between *E2F* expression and tumor stage in OC patients (GEPIA). Violin plot derived from correlation between the expression of a specific *E2F* family member and tumor stage in patients with OC; the *p*-value was set at 0.05. The distribution of *E2F1, E2F2, E2F3, E2F4, E2F5, E2F6, E2F7*, and *E2F8* mRNA expression correlated with tumor stage.

We compared the transcription expression of E2F family members between OC and normal tissues by using the GEPIA dataset (Figure 2). The results showed that the mRNA expression levels of *E2F1, E2F2, E2F3, E2F5*, and *E2F8* were significantly higher in OC tissues than in normal ovarian tissues, whereas the transcription expression levels of *E2F4, E2F6*, and *E2F7* were not significantly different between OC and normal tissues. By using the GEPIA dataset, we also analyzed the relationship between the transcription levels of *E2Fs* and the tumor stage of patients with OC. The mRNA expression of *E2F* family members was found to be significantly and negatively associated with the tumor stage for OC (Figure 3).

### Co-expression and Interaction Analyses of *E2F*s at the Gene and Protein Levels in Patients With OC

**Figure 4:**
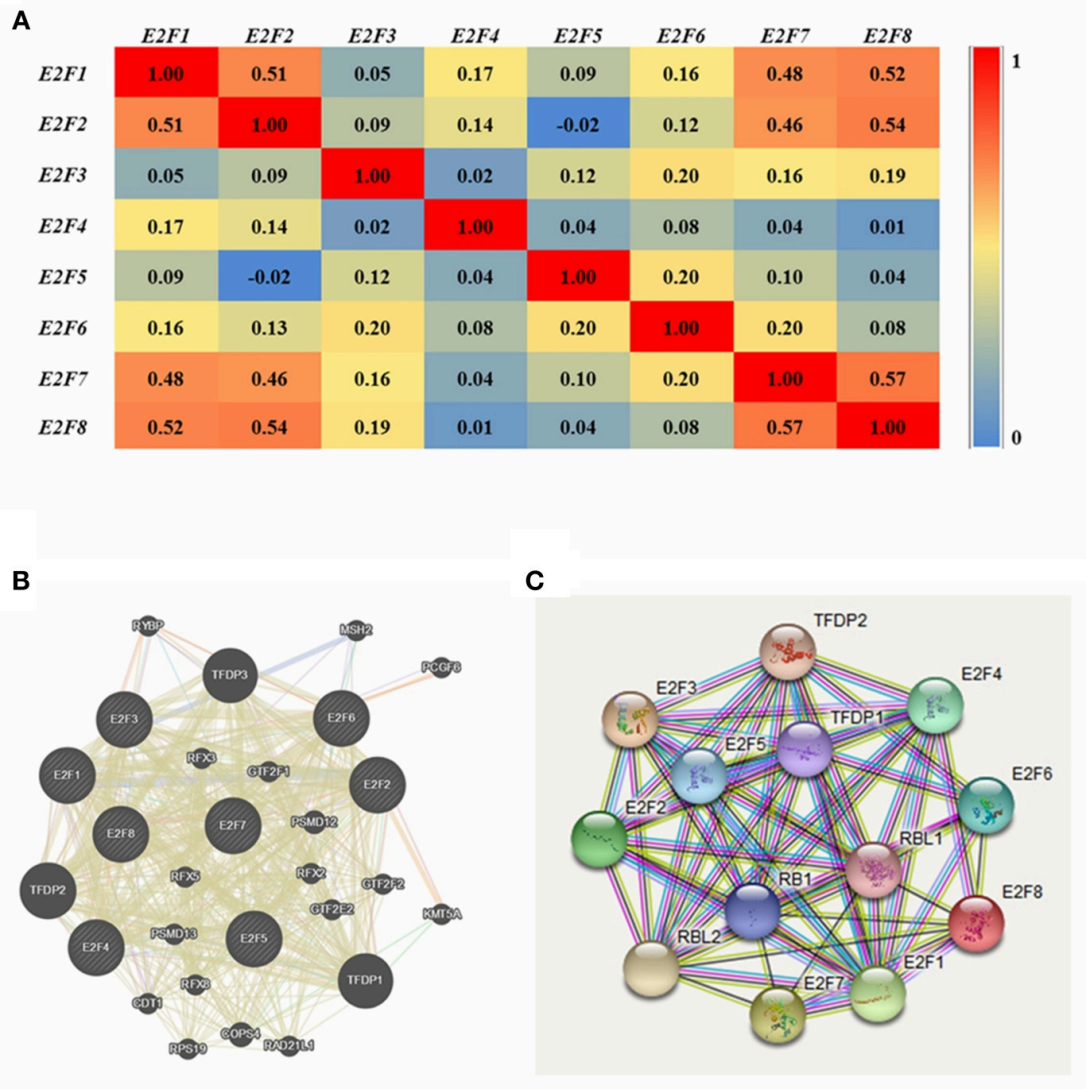
Co-expression and interaction analysis of *E2F* family members at the gene and protein levels in patients with OC (cBioPortal and GeneMANIA). **(A)** Pearson correlation of E2F family members. **(B)** Gene–gene interaction network among E2F family members in the cBioPortal dataset. **(C)** Protein–protein interaction network among E2F family members in the GeneMANIA dataset.

Pearson correlation analysis was conducted using expression data (*RNA Seq V2 RSEM*) of *E2F* family members collected from the cBioPortal online tool for OC (*TCGA*, Provisional). The results indicated a significant positive correlation among *E2F1* and *E2F3*; *E2F3* with *E2F1, E2F4*, and *E2F7*; *E2F4* with *E2F3* and *E2F8*; and *E2F8* with *E2F4*. However, significant negative correlations were noted for *E2F2* with *E2F5; E2F5* with *E2F2* and *E2F8;* and *E2F8* with *E2F2* (Figure 4A).

GeneMANIA was used to conduct correlation analysis of *E2F* family members at the gene level (Figure 4B). The results revealed relationships in co-expression between *E2F1* and *E2F2, E2F2* and *E2F5, E2F3* and *E2F2, E2F6* and *E2F5*, as well as *E2F7* and *E2F2*. Relationships were noted in co-localization between *E2F1* and *E2F3*, as well as *E2F3* and *E2F2*. Further, relationships were noted between E2F3 and E2F4 in genetic interactions, and *E2F1* and *E2F2* participated in a network group. In addition, the same pathway was shared between *E2F1* and *E2F3, E2F1*and *E2F4, E2F3* and *E2F4*, as well as *E2F4* and *E2F2*. Physical interactions were noted between *E2F1* and *E2F4* as well as *E2F7* and *E2F2*. Moreover, relationships were noted between *E2F1* with *E2F4* and *E2F5, E2F2* and *E2F5, E2F3* and *E2F4, E2F4* and *E2F2*, as well as *E2F6* and *E2F5*. Shared protein domains were noted among *E2F1* with *E2F5, E2F7*, and *E2F8*; *E2F2* with *E2F6* and *E2F8*; *E2F3* with *E2F5, E2F6, E2F7*, and *E2F8*; *E2F4* with *E2F5* and *E2F8*; *E2F6* and *E2F5*; *E2F7* with *E2F4, E2F5, E2F6*, and *E2F8*; as well as *E2F8* with *E2F5* and *E2F6*.

STRING analysis was conducted to identify interactions of *E2F* gene family members at the protein expression level. *E2F1* was shown to interact with *E2F2, E2F4*, and *E2F8*, and *E2F7* was found to interact with *E2F8* with regard to co-expression, text-mining, and protein homology. Detailed results are shown in Figure 4C.

### *E2F* Genetic Alteration and Neighbor Gene Network in Patients With OC

**Figure 5:**
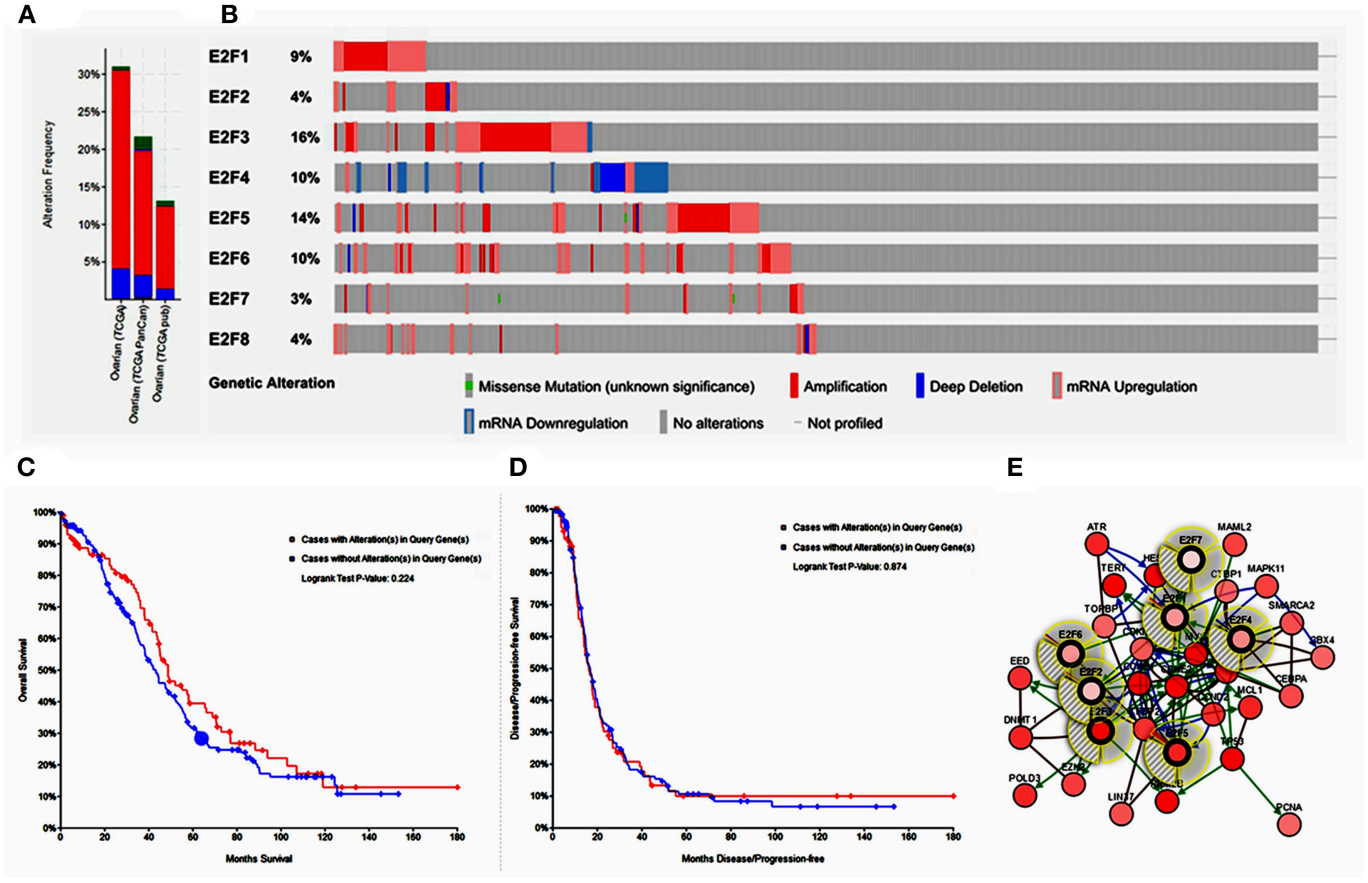
Alteration frequency of *E2F* family members and neighbor gene network in OC (cBioPortal). **(A)** Summary of alterations in *E2F* family members. **(B)** OncoPrint visual summary of alteration on a query of E2F family members. **(C)** Kaplan–Meier plots comparing OS in cases with/without *E2F* family member gene alterations. **(D)** Kaplan–Meier plots comparing disease-free survival (DFS) in cases with/without *E2F* family member alterations. **(E)** Gene–gene interaction network among *E2F* family members and 50 most frequently altered neighboring genes.

Alteration frequency of *E2F* mutations in OC was analyzed using cBioPortal. A total of 839 patients from three datasets of ovarian serous carcinoma were analyzed. Among the 3 OC datasets analyzed, gene set/pathway was altered in 389 (22.2%) of the queried samples, and alterations ranging from 31.02% (188/606) to 13.7% (83/606) were found for the gene sets submitted for analysis (Figure 5A). The percentages of genetic alterations in *E2F* family members for OC varied from 3 to 14% for individual genes based on the TCGA Provisional dataset (*E2F1*, 9%; *E2F2*, 4%; *E2F3*, 16%; *E2F4*, 10%; *E2F5*, 14%; *E2F6*, 10%; *E2F7*, 3%; E2F8, 4%; Figure 5B). The results of Kaplan–Meier plotter and log-rank test indicated no significant difference in OS and disease-free survival (DFS) between the cases with alterations in one of the query genes and those without alterations in any query genes (*P*-values, 0.224 and 0.874, respectively; Figures 5C,D). Next, we constructed the network for *E2Fs* and the 50 most frequently altered neighbor genes by using the cBioPortal. The results showed that *ATR, CBX4, CCND2, CCNE1, CCNE2, CDK6, CDKN1A, CDKN1B, CEBPA, CEEBBP, CTBP1, DNMT1, EED, EZH2, GSK3B, HDAC1, HES1, LIN37, LIN9, MAML2, MAMLD1, MAPK11, MCL1, MYC, NFATC2, PCNA, POLD3, POLG, PRMT5, RB1, RBBP4, RBL2, RPS6KB1, RRM2B, SMAD2, SMARCA2, SNW1, SUV39H1, SUZ12, TBP, TERT, TFDP2, TFE3, TK2, TOPBP1, TP53, TRRAP, UXT, XRCC1*, and *YY1* were closely associated with E2F alterations and functions (Figure 5E).

### Functional Enrichment Analysis of *E2Fs* in Patients With OC

**Figure 6:**
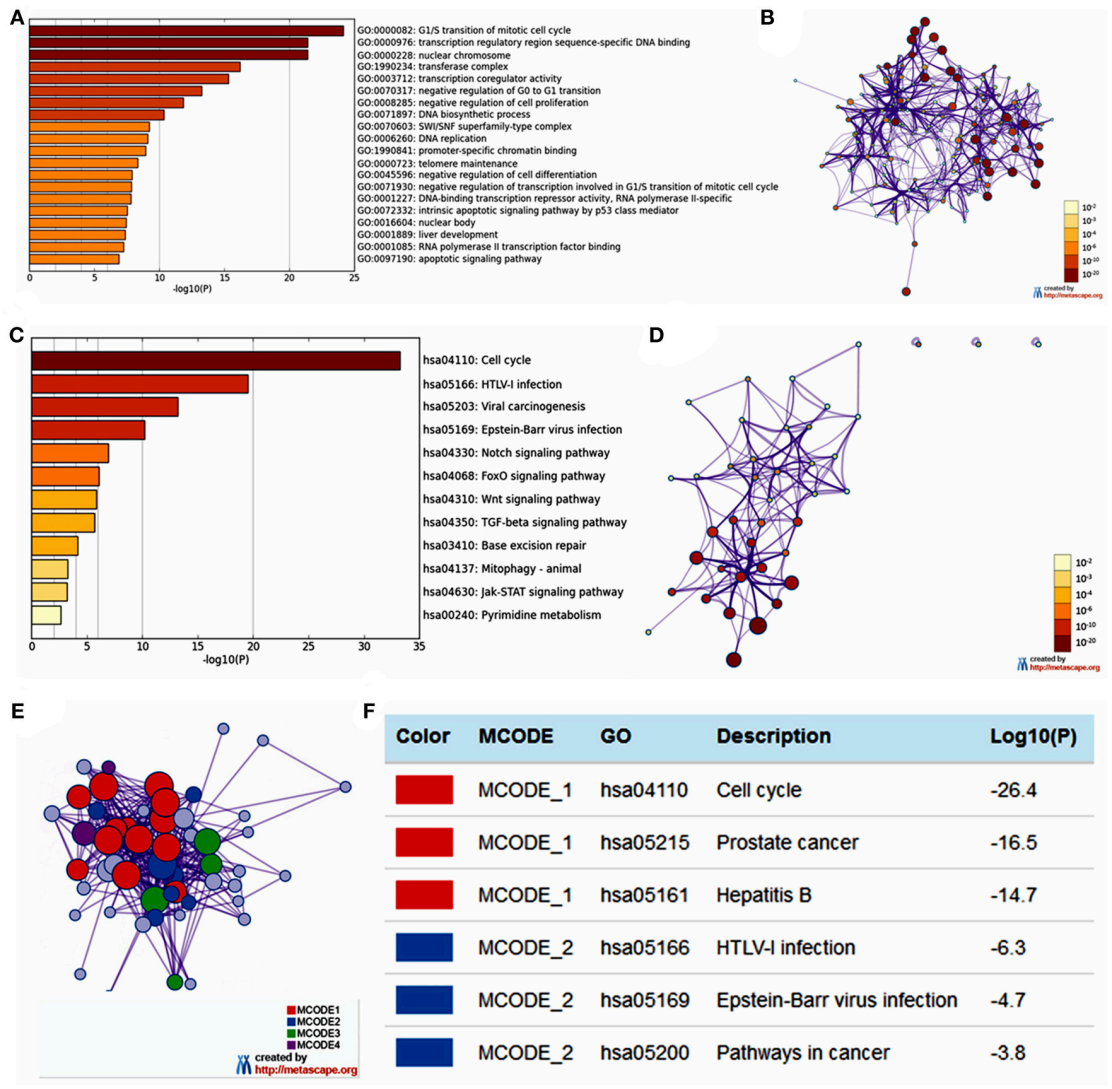
The enrichment analysis of *E2F* family members and neighboring genes in OC (Metascape). **(A)** Heatmap of Gene Ontology (GO) enriched terms colored by *p*-values. **(B)** Network of GO enriched terms colored by *p*-value, where terms containing more genes tend to have a more significant *p*-value. **(C)** Heatmap of Kyoto Encyclopedia of Genes and Genomes (KEGG) enriched terms colored by p-values. **(D)** Network of KEGG enriched terms colored by *p*-value, where terms containing more genes tend to have a more significant *p*-value. **(E)** Protein–protein interaction (PPI) network and four most significant MCODE components form the PPI network. **(F)** Independent functional enrichment analysis of three MCODE components.

The functions of *E2F* family members and their neighboring genes were predicted by analyzing GO and KEGG in Metascape. The top 20 GO enrichment items were classified into three functional groups: biological process group (11 items), molecular function group (5 items), and cellular component group (4 items; Figures 6A,B and Table 2). The *E2F* family members and their neighboring genes were mainly enriched in cell cycle, apoptosis, and transcriptional regulation biological processes such as G1/S transition of mitotic cell cycle, negative regulation of G0 to G1 transition, negative regulation of cell proliferation, DNA biosynthetic process, DNA replication, telomere maintenance, negative regulation of cell differentiation, negative regulation of transcription involved in G1/S transition of mitotic cell cycle, intrinsic apoptotic signaling pathway by p53 class mediator, liver development, and apoptotic signaling pathway. The molecular functions for these genes were mainly transcription regulation by sequence-specific DNA binding, transcription co-regulator activity, promoter-specific chromatin binding, DNA-binding transcription repressor activity, RNA polymerase II-specific, and RNA polymerase II transcription factor binding; the cellular components that these genes were involved in were the nuclear chromosome, transferase complex, SWI/SNF superfamily-type complex, and nuclear body.

**Table 2:** The GO function enrichment analysis of E2F family members and neighbor genes in OC (GeneMANIA).

**GO**	**Category**	**Description**	**Count**	**%**	**Log_**10**_(*P*)**	**Log_**10**_(*q*)**
GO:0000082	GO biological processes	G1/S transition of mitotic cell cycle	20	35.09	−24.16	−19.82
GO:0070317	GO biological processes	Negative regulation of G0 to G1 transition	8	14.04	−13.25	−10.65
GO:0008285	GO biological processes	Negative regulation of cell proliferation	17	29.82	−11.84	−9.31
GO:0071897	GO biological processes	DNA biosynthetic process	10	17.54	−10.36	−7.85
GO:0006260	GO biological processes	DNA replication	10	17.54	−9.09	−6.60
GO:0000723	GO biological processes	Telomere maintenance	8	14.04	−8.35	−5.89
GO:0045596	GO biological processes	Negative regulation of cell differentiation	13	22.81	−7.90	−5.48
GO:0071930	GO biological processes	Regulation of transcription involved in G1/S transition of mitotic cell cycle	3	5.26	−7.85	−5.44
GO:0072332	GO Biological processes	Intrinsic apoptotic signaling pathway by p53 class mediator	6	10.53	−7.53	−5.13
GO:0001889	GO biological processes	Liver development	7	12.28	−7.38	−5.01
GO:0097190	GO biological processes	Apoptotic signaling pathway	11	19.30	−6.89	−4.57
GO:0000228	GO cellular components	Nuclear chromosome	23	40.35	−21.41	−17.68
GO:1990234	GO cellular components	Transferase complex	21	36.84	−16.20	−13.23
GO:0070603	GO cellular components	SWI/SNF superfamily-type complex	7	12.28	−9.24	−6.74
GO:0016604	GO cellular components	Nuclear body	13	22.81	−7.47	−5.08
GO:0000976	GO molecular functions	Transcription regulatory region sequence-specific DNA binding	25	43.86	−21.41	−17.68
GO:0003712	GO molecular functions	transcription coregulator activity	18	31.58	−15.31	−12.52
GO:1990841	GO molecular functions	Promoter-specific chromatin binding	6	10.53	−8.96	−6.48
GO:0001227	GO molecular functions	DNA-binding transcription repressor activity, RNA polymerase II-specific	9	15.79	−7.82	−5.40
GO:0001085	GO molecular functions	RNA polymerase II transcription factor binding	7	12.28	−7.25	−4.90

The top 12 KEGG pathways for the *E2F* family members and their neighboring genes are shown in Figures 6C,D and Table 3. Among these pathways, the cell cycle signaling pathway, viral carcinogenesis signaling pathway, TGF-beta signaling pathway, Wnt signaling pathway, and Jak-STAT signaling pathway were found to be related to multiple tumor development and were involved in OC tumorigenesis and pathogenesis. In addition, to better understand the relationship between E2F family members and OC, we performed a Metascape protein–protein interaction enrichment analysis. The protein–protein interaction network and MCODE components identified in the gene lists are shown in Figures 6E,F. The four most significant MCODE components were extracted from the protein–protein interaction network. After pathway and process enrichment analysis was independently applied to each MCODE component, the results showed that biological function was mainly related to cell cycle, prostate cancer, hepatitis B, HTLV-I infection, Epstein–Barr virus infection, and pathways in cancer.

**Table 3:** The KEGG function enrichment analysis of E2F family members and neighbor genes in OC (GeneMANIA).

**GO**	**Category**	**Description**	**Count**	**%**	**Log_**10**_(P)**	**Log_**10**_(q)**
hsa04110	KEGG pathway	Cell cycle	21	36.84211	−33.2463	−30.5526
hsa05166	KEGG pathway	HTLV-I infection	17	29.82456	−19.547	−17.1543
hsa05203	KEGG pathway	Viral carcinogenesis	12	21.05263	−13.2006	−11.5068
hsa05169	KEGG pathway	Epstein-Barr virus infection	10	17.54386	−10.2055	−8.68789
hsa04330	KEGG pathway	Notch signaling pathway	5	8.77193	−6.93209	−5.58078
hsa04068	KEGG pathway	FoxO signaling pathway	6	10.52632	−6.06809	−4.73609
hsa04310	KEGG pathway	Wnt signaling pathway	6	10.52632	−5.86461	−4.55109
hsa04350	KEGG pathway	TGF-beta signaling pathway	5	8.77193	−5.70524	−4.40946
hsa03410	KEGG pathway	Base excision repair	3	5.263158	−4.1406	−2.90927
hsa04137	KEGG pathway	Mitophagy—animal	3	5.263158	−3.26134	−2.11707
hsa04630	KEGG pathway	Jak-STAT signaling pathway	4	7.017544	−3.23343	−2.10791
hsa00240	KEGG pathway	Pyrimidine metabolism	3	5.263158	−2.65879	−1.59853

### Prognostic Values of *E2Fs* in Patients With OC

**Figure 7:**
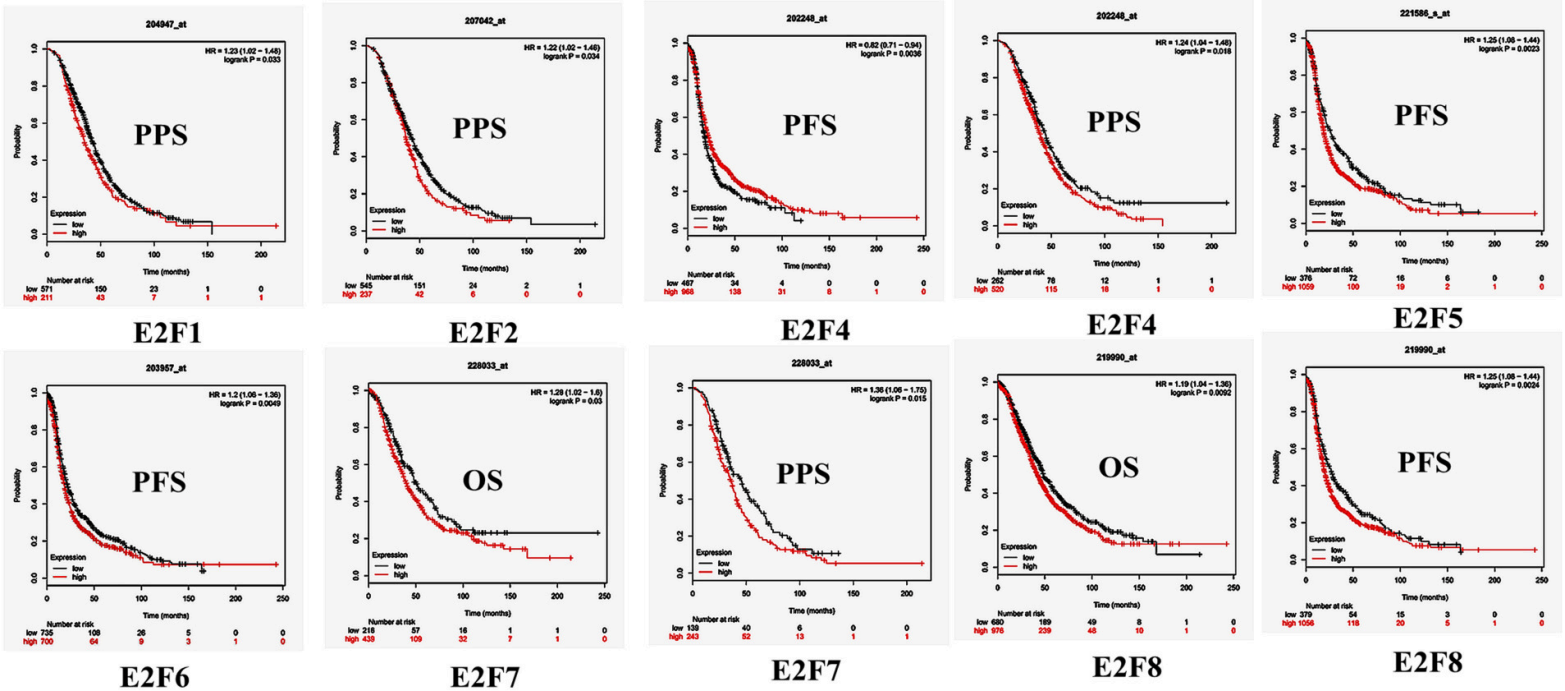
The prognostic value of mRNA level of *E2F* family members in OC patients (Kaplan–Meier plotter). The OS, PFS, and PPS survival curves comparing patients with high (red) and low (black) E2F family member expression in OC were plotted using Kaplan–Meier plotter database at the threshold of *p*-value of < 0.05.

By using Kaplan–Meier plotter analysis, we initially assessed the prognostic significance of the *E2F* family members in all OC patients. The Kaplan–Meier survival curves are shown in Figure 7 and Table 4. The increased mRNA levels of *E2F7* and *E2F8* were strongly associated with poor OS; the remaining *E2F* family members were not related with OS in OC. The high expression of *E2F5, E2F6*, and *E2F8* mRNA was predicted to have worse PFS, whereas high *E2F4* mRNA expression was correlated to longer PFS in OC patients. In addition, increased *E2F1, E2F2, E2F4*, and *E2F7* mRNA expression levels were associated with poor PPS.

**Table 4:** The Prognostic values of *E2F* family members in all and different pathological subtypes OC patients (Kaplan-Meier plotter).

**E2F family**	**Histology**	**OS**				**PFS**				**PPS**			
		**Cases**	**HR**	**95% CI**	***p*-value**	**Cases**	**HR**	**95% CI**	***p*-value**	**Cases**	**HR**	**95% CI**	***p*-value**
E2F1	Overall	1,656	1.10	0.96–1.25	0.16	1,435	0.91	0.80–1.04	0.17	**782**	**1.23**	**1.02**–**1.48**	**0.03**
204947_at	Serous	**1,207**	**1.18**	**1.00**–**1.39**	**0.05**	1,104	1.06	0.90–1.26	0.47	**735**	**1.22**	**1.01**–**1.49**	**0.04**
	Endometrioid	37	—	—	0.07	**51**	**6.12**	**1.4**–**26.69**	**0.01**	14	—	—	—
E2F2	Overall	1,656	0.90	0.79–1.02	0.10	1,435	0.88	0.78–1	0.06	**782**	**1.22**	**1.02**–**1.46**	**0.03**
207042_at	Serous	**1,207**	**0.80**	**0.68**–**0.95**	**0.01**	**1,104**	**1.17**	**1**–**1.36**	**0.05**	**735**	**1.20**	**1**–**1.45**	**0.05**
	Endometrioid	37			0.16	**51**	**0.33**	**0.12**–**0.9**	**0.02**	14	—	—	—
E2F3	Overall	1,656	1.11	0.97–1.26	0.12	1,435	1.08	0.93–1.25	0.32	782	1.13	0.95–1.33	0.17
203692_s_at	Serous	**1,207**	**1.18**	**1.00**–**1.39**	**0.05**	1,104	0.89	0.77–1.03	0.13	**735**	**1.20**	**1**–**1.42**	**0.05**
	Endometrioid	37	2.68	0.45–16.08	0.26	**51**	**8.63**	**1.15**–**64.93**	**0.01**	14	—	—	—
E2F4	Overall	1,656	0.93	0.81–1.07	0.31	**1,435**	**0.82**	**0.71**–**0.94**	**0.00**	**782**	**1.24**	**1.04**–**1.48**	**0.02**
202248_at	Serous	1,207	0.92	0.78–1.07	0.28	**1,104**	**1.20**	**1.03**–**1.4**	**0.02**	**735**	**1.21**	**1.01**–**1.45**	**0.04**
	Endometrioid	37	—	—	—	51	2.01	0.75–5.36	0.15	14	—	—	—
E2F5	Overall	1,656	1.10	0.96–1.25	0.16	**1,435**	**1.25**	**1.08**–**1.44**	**0.00**	782	0.85	0.7–1.02	0.08
221586_s_at	Serous	1,207	0.92	0.79–1.08	0.31	1,104	0.95	0.82–1.09	0.45	735	0.86	0.71–1.05	0.14
	Endometrioid	37	0.33	0.06–1.99	0.20	**51**	**0.32**	**0.12**–**0.81**	**0.01**	14	—	—	—
E2F6	Overall	1,656	1.12	0.96–1.3	0.14	**1,435**	**1.20**	**1.06**–**1.36**	**0.00**	782	0.85	0.71–1.01	0.06
203957_at	Serous	1,207	0.90	0.77–1.06	0.21	1,104	1.06	0.92–1.23	0.43	**735**	**0.82**	**0.69**–**0.98**	**0.03**
	Endometrioid	**37**	**6.63**	**0.74**–**59.46**	**0.05**	51	1.44	0.55–3.78	0.46	14	—	—	—
E2F7	Overall	**655**	**1.28**	**1.02**–**1.6**	**0.03**	614	1.12	0.93–1.35	0.24	**382**	**1.36**	**1.06**–**1.75**	**0.02**
228033_at	Serous	**523**	**1.31**	**1.04**–**1.64**	**0.02**	483	0.88	0.7–1.11	0.27	**346**	**1.35**	**1.04**–**1.76**	**0.03**
	Endometrioid	30	—	—	0.13	51	0.41	0.14–1.19	0.09	10	—	—	—
E2F8	Overall	**1,435**	**1.19**	**1.04**–**1.61**	**0.02**	**1,435**	**1.25**	**1.08**—**1.44**	**0.00**	782	1.15	0.96–1.38	0.12
219990_at	Serous	**1,207**	**1.24**	**1.04**–**1.48**	**0.02**	1,104	0.88	0.76–1.01	0.07	735	1.18	0.98–1.42	0.09
	Endometrioid	37	—	—	—	**51**	**2.82**	**1.00**–**7.96**	**0.04**	14	—	—	—

*The bold values indicate that the results are statistically significant*.

Further, we assessed the correlation of individual *E2F* family members with different pathological and histological subtypes of OC, including serous and endometrioid. The high mRNA expression levels of *E2F1, E2F3, E2F7*, and *E2F8* were correlated to poor OS in serous OC patients, whereas increased *E2F2* mRNA expression was associated with better OS in serous OC patients. Further, increased *E2F2* and *E2F4* mRNA expression levels were associated with poor PFS in serous OC patients. High mRNA expression of *E2F1, E2F2, E2F3, E2F4*, and *E2F7* was significantly associated with worse PPS. However, increased E2F6 mRNA expression level was correlated with better PPS. In endometrioid OC, none of the *E2F* family members were related with prognosis in endometrioid OC. High *E2F1, E2F3*, and *E2F8* mRNA expression levels were associated with poor PFS, whereas increased *E2F2* and *E2F5* mRNA expression levels were associated with superior PFS in endometrioid OC patients. Data to calculate PPS in patients with endometrial OC based on Kaplan–Meier online tool were not sufficient. The prognostic value of mRNA level of E2F family members in OC patients using Kaplan–Meier plotter (*p* > 0.05) are shown in Appendix.

## Discussion

Numerous studies have suggested that *E2Fs* are involved in not only proliferation and differentiation but also apoptosis and tumorigenesis (7, 10). Accumulative evidence indicated that aberrant expression or activation of *E2Fs* is a common phenomenon in malignances, and significant associations between *E2Fs* and tumorigenesis or progression of patients with cancer has been partially confirmed (10, 29). However, the patterns of expression and the exact roles of distinct *E2F* family members in OC are not yet known. In this study, we attempted to systematically explore the expression patterns, prognostic values, genetic alteration, correlation, and potential functions of different *E2Fs* in OC.

*E2F1*, the most classic member of the *E2F* family, was found to play roles in both proliferation and apoptosis and exhibited a complex role in tumor development regulation (30). *E2F1* has been shown sto exhibit dual properties and can act as a tumor suppressor or oncogene in the same cancer (11, 13, 31). However, E2F1 overexpression is known to contribute to the development and progression of OC, and this role is mediated by the p53-dependent apoptotic pathway and PI3K/AKT signaling pathway and microRNA activity (13, 31). More and more studies revealed that E2F1 overexpression could produce more aggressive tumors with a high proliferation rate and chemoresistance (32–34). In our study, ONCOMINE and GEPIA datasets revealed that the expression of *E2F1* was up-regulated in human OC, and *E2F1* expression was linked with the clinical characteristics of patients with OC. By using Kaplan–Meier plotter, we found increased *E2F1* RNA expression level, which was associated with poor PPS in all patients with OC, which seemed consistent with the role of E2F3 as an oncogene.

*E2F2* regulates many cell processes such as cell cycle, DNA synthesis, proliferation, and tumorigenesis (35). Previous studies indicated that like *E2F1, E2F2* exhibited oncogenic or tumor suppressive activity, and overexpression of *E2F2* contributed to the development of several solid tumors, indicating worse patient outcome (36, 37). However, the predictive roles of E2F2 for oncogenesis, prognosis, and prediction of therapeutic in human OC are not yet completely understood. In this study, *E2F2* expression was found to be higher in OC tissues than in normal tissues and was significantly and negatively correlated with tumor stage in patients with OC. In addition, high *E2F2* expression was significantly correlated with worse PPS in all patients with OC.

The *E2F3* transcription factor is known to play a role in controlling cell cycle progression. Recently, the clear oncogenic role of *E2F3* was revealed in several human cancers (38). Amplification and overexpression of *E2F3* has been shown to be closely associated with clinical stage, pathological grading, proliferation index, and tumor aggression (39). Interestingly, *E2F3a* was found to be essential in EGFR-mediated proliferation in ovarian cancer cells (40). An *in vitro* study showed that siRNA for *E2F3* facilitated the silencing of *E2F3* overexpression and protected against breast cancer. Therefore, *E2F3* might be a newly identified diagnostic and potential therapeutic target for solid human cancers (41). In this study, the expression of *E2F3* in OC tissues was higher than that in normal tissues. We also found that *E2F3* expression was significantly and negatively correlated with tumor stage in patients with OC. Further, although no significant association was observed between E2F3 and clinical outcomes in all OC patients, subgroup analysis revealed that *E2F3* overexpression was associated with reduced OS and PPS in serous OC patients, as well as worse PFS in endometrioid OC patients.

*E2F4* is a key regulator of cell transformation, proliferation, and cell cycle progression, and a recent study showed that patients exhibiting high expression of *E2F4* target genes exhibited more severe cancer and shorter survival (42). In OC, *E2F4* is involved in cell cycle arrest at the G0 phase in *TOV21G* and *SKOV3* cells, and this role is enhanced by deregulated cyclin-dependent kinase inhibitors such as p27, p130/Rb2, and p130/Rb2 (43). Lawrenson et al. (44) confirmed that *E2F4* variants are associated with OC pathogenesis by conducting genome-wide association studies. Reimer et al. (14, 15) found that the expression level of *E2F4* was lower in tissues of platinum-resistant OC patients than in tissues of platinum-sensitive patients, which indicated a tumor suppressor function and prognostic value for *E2F4*. In our study, the expression of *E2F4* was slightly lower in OC tissues than in normal ones and was markedly and negatively correlated with tumor stage in patients with OC. Survival analysis results showed that increased *E2F4* expression was significantly correlated with longer PFS in all OC patients.

*E2F5* is an important member of the E2F family. It has growth-repressive characteristics that have been observed in several solid cancers such as osteosarcoma, colon cancer, breast cancer, and OC (4). A recent study showed *E2F5* overexpression in early as well as advanced stages of OC, and *E2F5* status was shown to significantly improve malignancy diagnosis of epithelial OC (12). Moreover, silencing of *E2F5* by using miR-132 inhibited the proliferation, colony formation, migration, and invasion of OC cells (45). Thus, *E2F5* has been suggested to have a putative role in OC pathogenesis. In this study, *E2F5* expression was higher in OC tissues than in normal ones and was significantly and negatively correlated with tumor stage in patients with OC. Furthermore, an elevated level of *E2F5* was significantly associated with a worse PFS in all patients with OC.

*E2F6*, one of the unique E2F family members, is known to be a pRb-independent transcription repressor of *E2F*-target genes (46). Although the possible links between *E2F6* and cell growth control are intriguing, little is known about the regulation mechanism, and the expression pattern and prognostic role of *E2F6* in OC are not yet known. In this study, no significant difference in *E2F6* expression was noted between OC tissues and normal ones, but *E2F6* expression was negatively correlated with tumor stage in patients with OC. Interestingly, the overexpression of *E2F6* was significantly correlated with worse PFS in all patients with OC.

*E2F7* is an atypical *E2F* family member that acts as a transcriptional repressor of E2F target genes, thereby contributing to cell cycle arrest for DNA repair and genomic integrity (47). One study showed that the down-regulation of *E2F7* might contribute to platinum resistance, and high expression of *E2F7* predicted favorable DFS and OS in OC (14). Clements et al. (48) revealed that BRCA2-deficient cells are less sensitive to PARP inhibitor and cisplatin treatment after *E2F7* depletion, thereby indicating that *E2F7* could be a putative biomarker for tumor response to PARP inhibitor therapy. In this study, like that of *E2F6*, no significant difference in *E2F7* expression was noted between OC and normal tissues, but *E2F7* expression was significantly and negatively correlated with tumor stage in patients with OC. Further, high *E2F7* expression was significantly correlated with poor PFS and PPS in all and serous OC patients, indicating its oncogenic role in OC.

*E2F8* is a recently identified member of the *E2F* family with a duplicated DNA-binding domain feature discriminated from that in *E2F1-6* (49). Accumulating evidence indicates that E2F8 is indispensable for angiogenesis, lymphangiogenesis, and embryonic development. *E2F8* is highly expressed in various tumors and promotes tumor progression, and serves as a therapeutic target in lung and liver cancers (50, 51). Unfortunately, there is little research evidence between *E2F8* and ovarian cancer diagnosis, staging, prognosis, and targeted drug therapy. In this study, *E2F8* was significantly overexpressed in OC tissues, and its expression was markedly and negatively correlated with the tumor stage of patients with OC. Interestingly, high *E2F8* expression was significantly correlated with poor OS and PFS in all patients with OC.

Growing evidence suggests that the cross-talk of the eight members of the *E2F* family is causatively involved in cell cycle control, cell proliferation, apoptosis, and carcinogenesis (8, 9, 31, 52). In this study, co-expression and correlation analyses of the E2F family were performed at both the gene and protein levels. These findings are similar to those of previous studies. For example, *E2F1* and *E2F3* were shown to be target genes involved in the *p53* and *p73* pathways for inducing apoptosis in a transgenic mouse model (53). Reimer et al. (14, 15) showed that deregulation of both proliferation-promoting and proliferation-inhibiting *E2F* transcription factors and their cross-talk is crucial for tumor progression of OC and influence clinical outcome; thus, they could be possible useful targets in anti-cancer therapy. Although we partially recognized the important role of *E2F* interactions in the pathogenesis and development of OC, the cross-talk and specific molecular mechanisms of *E2F* family members remain to be further investigated.

To further clarify the genetic alteration, potential function, and carcinogenic mechanism of the *E2F* family members, we calculated the percentages of genetic alterations in *E2F* family members for OC and found that they varied from 3 to 14% for individual genes based on TCGA Provisional dataset. Further, cases with alterations in one of the query gene had worse OS and DFS than those without any alterations in the query genes, but the difference was not statistically significant. We constructed a network for E2F family members and 50 neighboring genes. The results of functional analysis indicated that these genes are mainly enriched in tumor-related pathways related to the development of multiple tumors. Our study adds to the growing evidence regarding the complexity of the *E2F* family members and their associated signaling pathways, which offer clues into the rational development of multi-targeted and E2F-mediated targeted therapy.

## Conclusions

In summary, our results indicated that the mRNA expression levels of *E2F1, E2F3, E2F5*, and *E2F8* were significantly upregulated, and obvious and negatively associated with tumor stage for OC. Furthermore, aberrant expression of *E2F2, E2F5, E2F7*, and *E2F8* were found to be associated with the clinical outcomes of patients with OC. These results suggest that *E2F2, E2F5, and E2F8* may serve as potential prognostic biomarkers and targets for OC. These results may be beneficial to better understand the molecular underpinning of OC and may be useful to develop tools for more accurate OC prognosis and for promoting the development of *E2F*-mediated drug for OC treatment. However, future studies are required to validate our findings and thus promote the clinical utility of E2Fs serving as prognostic indicators or therapeutic targets in OC.

## Author Contributions

QZ and M-ZZ developed the idea and designed the research. FZ and ZH analyzed the data. QZ and ZH wrote the draft of the manuscript. QZ and M-ZZ obtained copies of studies and revised the writing. All authors read and approved the submitted version.

### Conflict of Interest Statement

The authors declare that the research was conducted in the absence of any commercial or financial relationships that could be construed as a potential conflict of interest.

## Supplementary Material

Appendix The prognostic value of mRNA level of E2F family members in OC patients using Kaplan-Meier plotter (*p* > 0.05).

## Abbreviations

OC: ovarian cancer
EGFR: epidermal growth factor receptor
GEPIA: Gene Expression Profiling Interactive Analysis
DAVID: The Database for Annotation, Visualization and Integrated Discovery
GO: Gene Ontology
KEGG: Kyoto Encyclopedia of Genes and Genomes
OS: overall survival
PFS: progression-free survival
PPS: post progression survival
DFS: disease-free survival.
