# Supplementary figures and images for: E2F2/5/8 Serve as Potential Prognostic Biomarkers and Targets for Human Ovarian Cancer
Source: Front Oncol. 2019 Mar 22;9:161. doi: 10.3389/fonc.2019.00161 (PMC6439355; doi:10.3389/fonc.2019.00161)

# 204947\_at

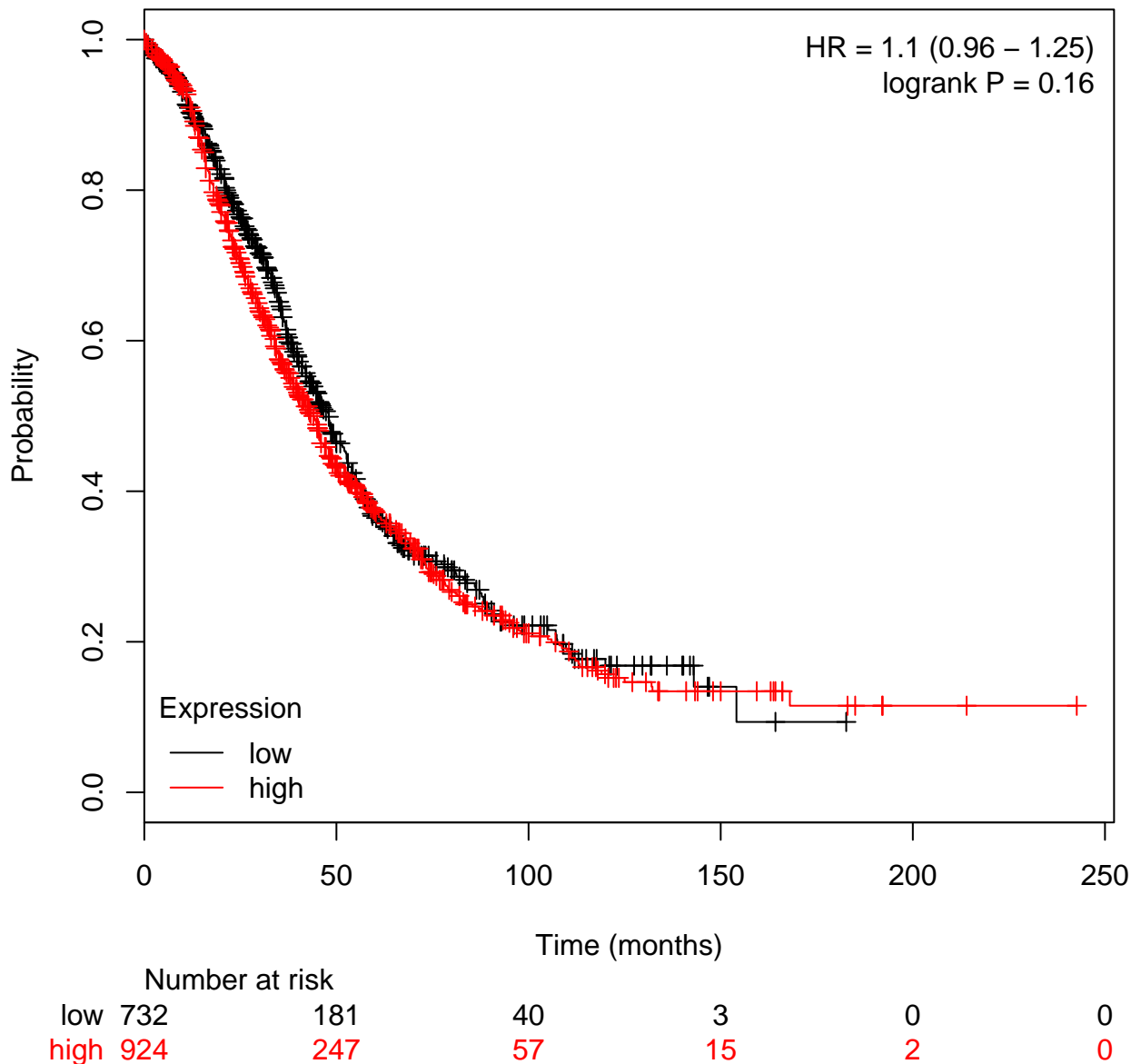

Supplement: Appendix — The prognostic value of mRNA level of E2F family members in OC patients using Kaplan-Meier plotter (p > 0.05). [file Data_Sheet_1.zip › E2F1-OS_204947_at.pdf]

## 204947\_at

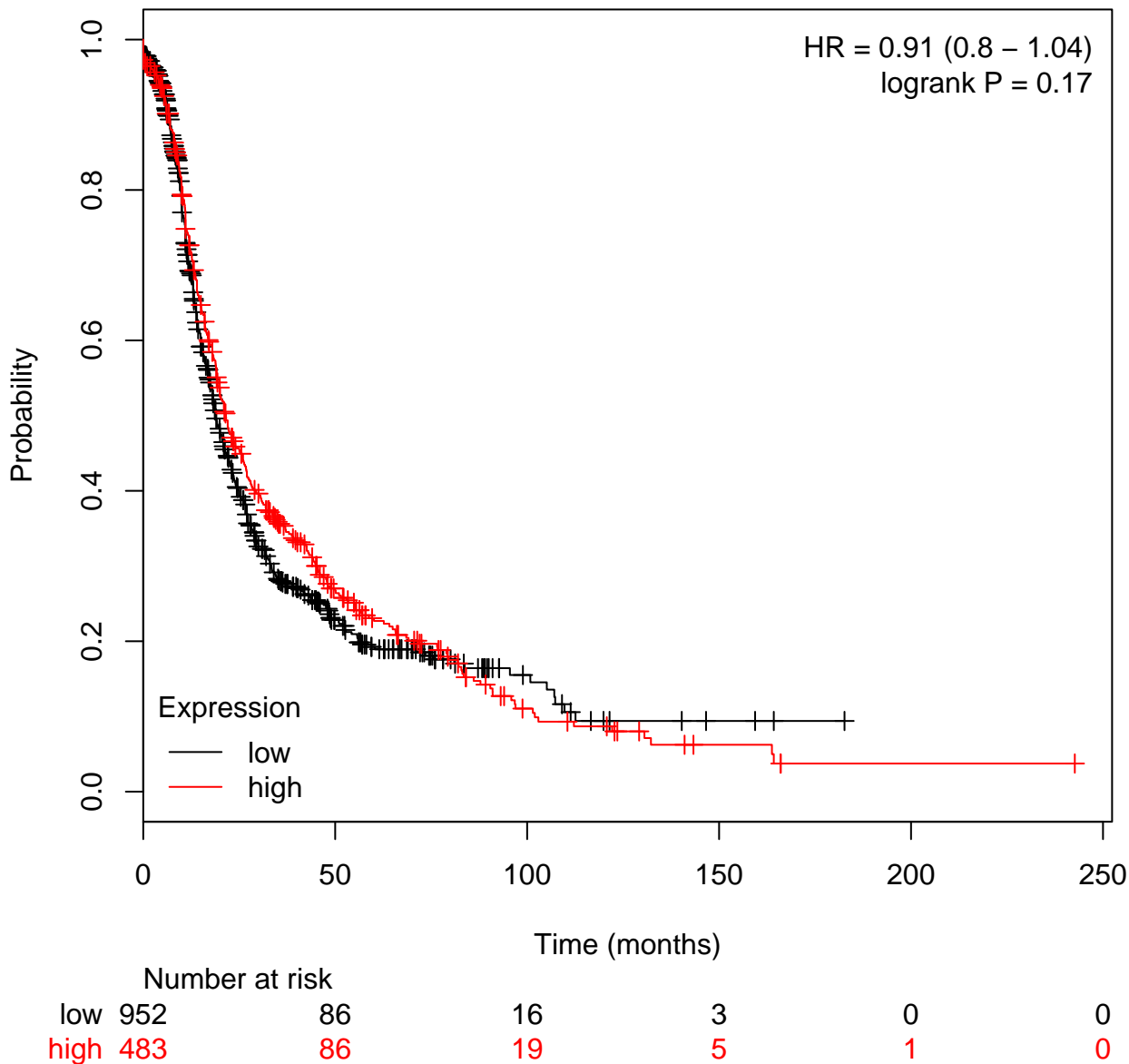

Supplement: Appendix — The prognostic value of mRNA level of E2F family members in OC patients using Kaplan-Meier plotter (p > 0.05). [file Data_Sheet_1.zip › E2F1-PFS_204947_at.pdf]

## 207042\_at

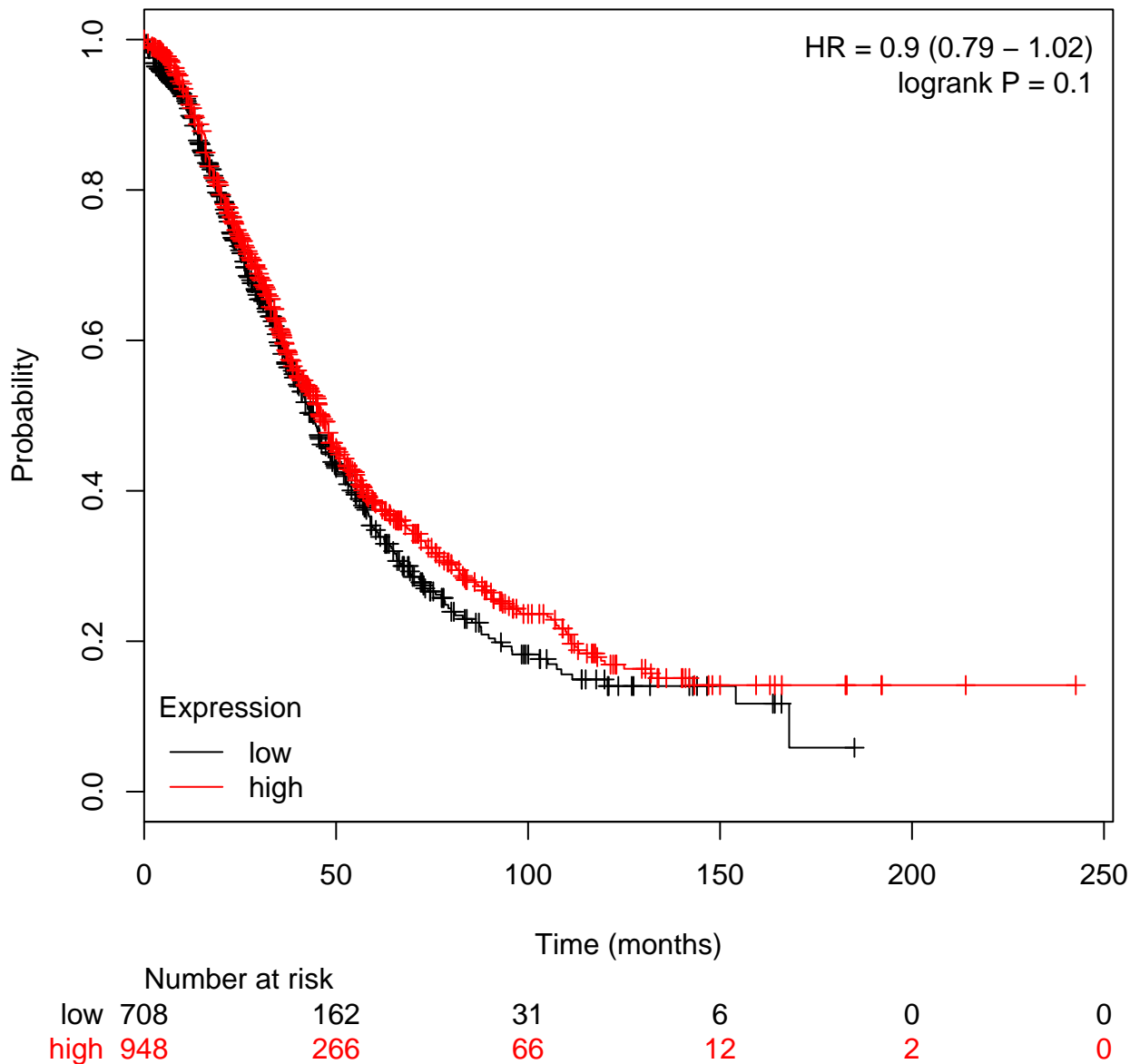

Supplement: Appendix — The prognostic value of mRNA level of E2F family members in OC patients using Kaplan-Meier plotter (p > 0.05). [file Data_Sheet_1.zip › E2F2-OS_207042_at.pdf]

## 207042\_at

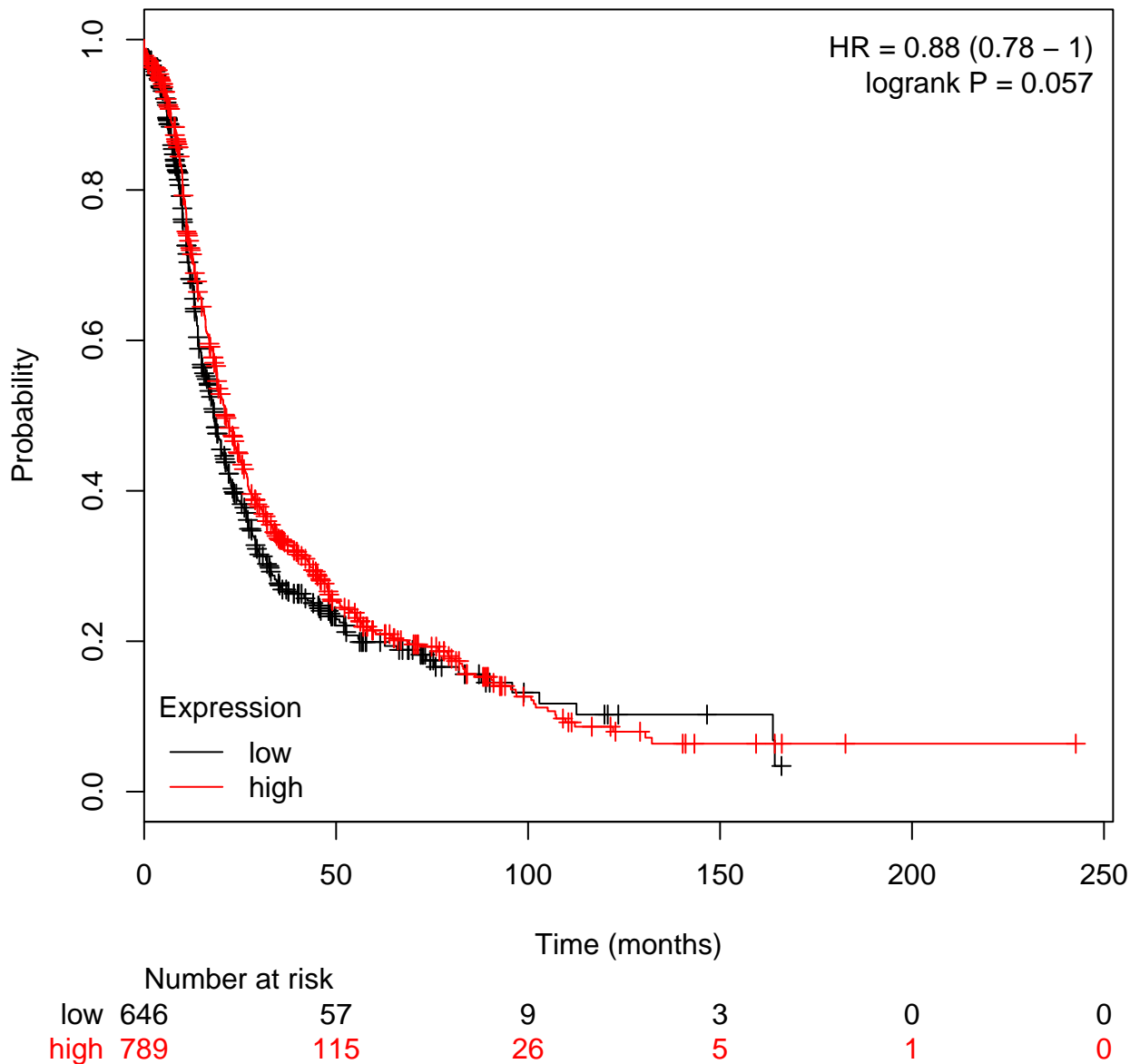

Supplement: Appendix — The prognostic value of mRNA level of E2F family members in OC patients using Kaplan-Meier plotter (p > 0.05). [file Data_Sheet_1.zip › E2F2-PFS_207042_at.pdf]

# 203692\_s\_at

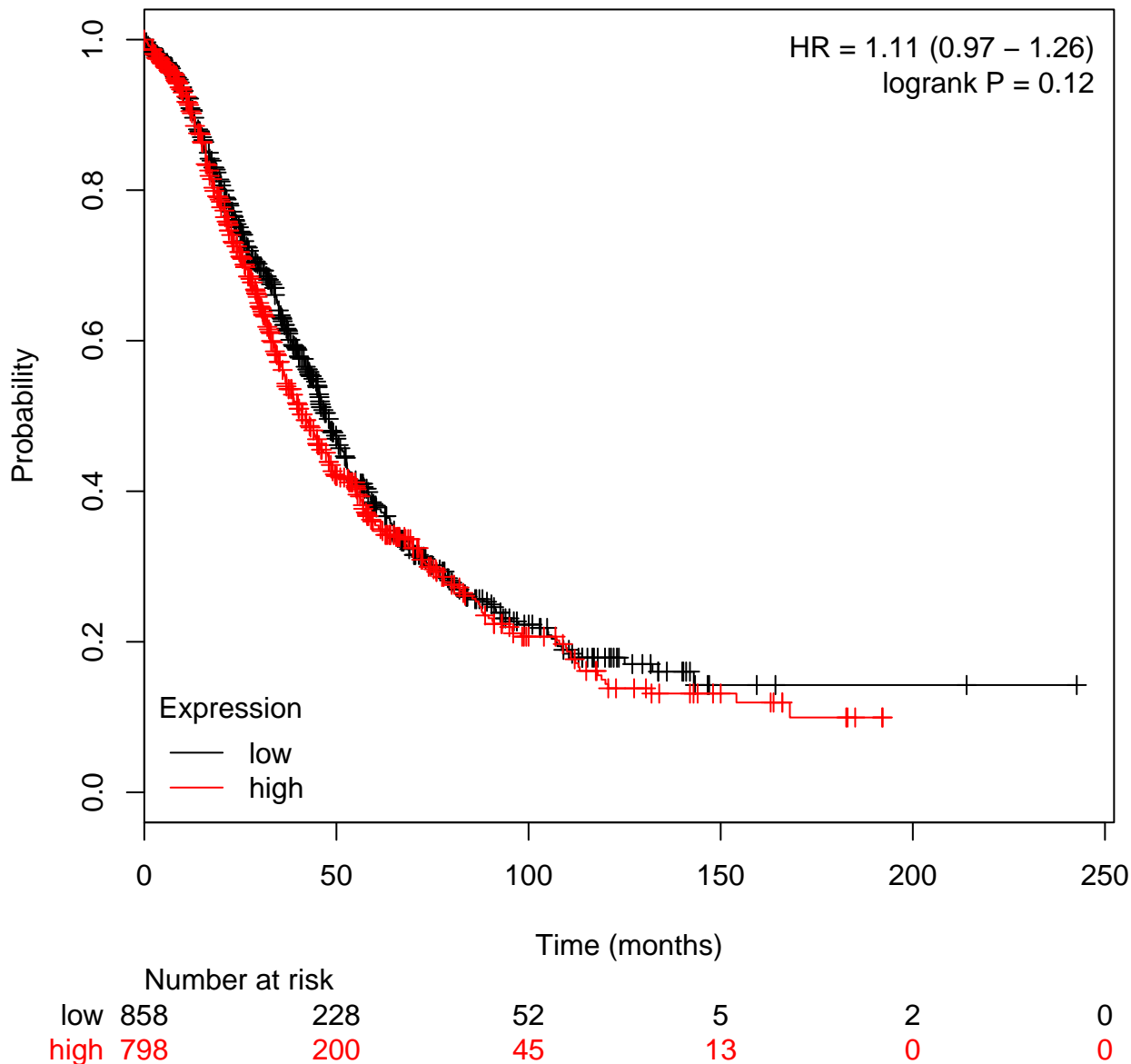

Supplement: Appendix — The prognostic value of mRNA level of E2F family members in OC patients using Kaplan-Meier plotter (p > 0.05). [file Data_Sheet_1.zip › E2F3-OS_203692_s_at.pdf]

# 203692\_s\_at

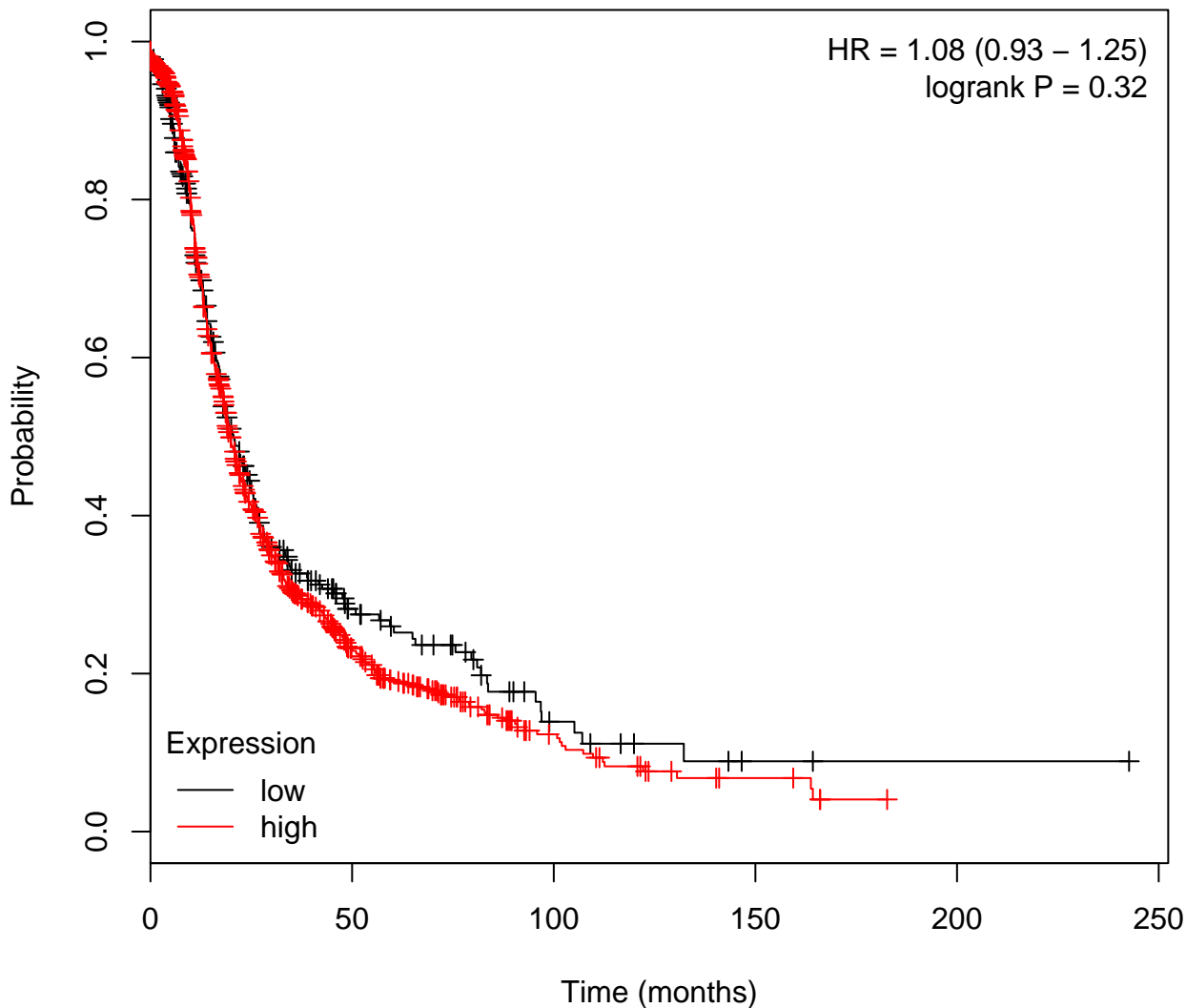

Number at risk

|      |      |     |    |   |   |   |
|------|------|-----|----|---|---|---|
| low  | 358  | 40  | 10 | 2 | 1 | 0 |
| high | 1077 | 132 | 25 | 6 | 0 | 0 |

Supplement: Appendix — The prognostic value of mRNA level of E2F family members in OC patients using Kaplan-Meier plotter (p > 0.05). [file Data_Sheet_1.zip › E2F3-PFS_203692_s_at.pdf]

# 203692\_s\_at

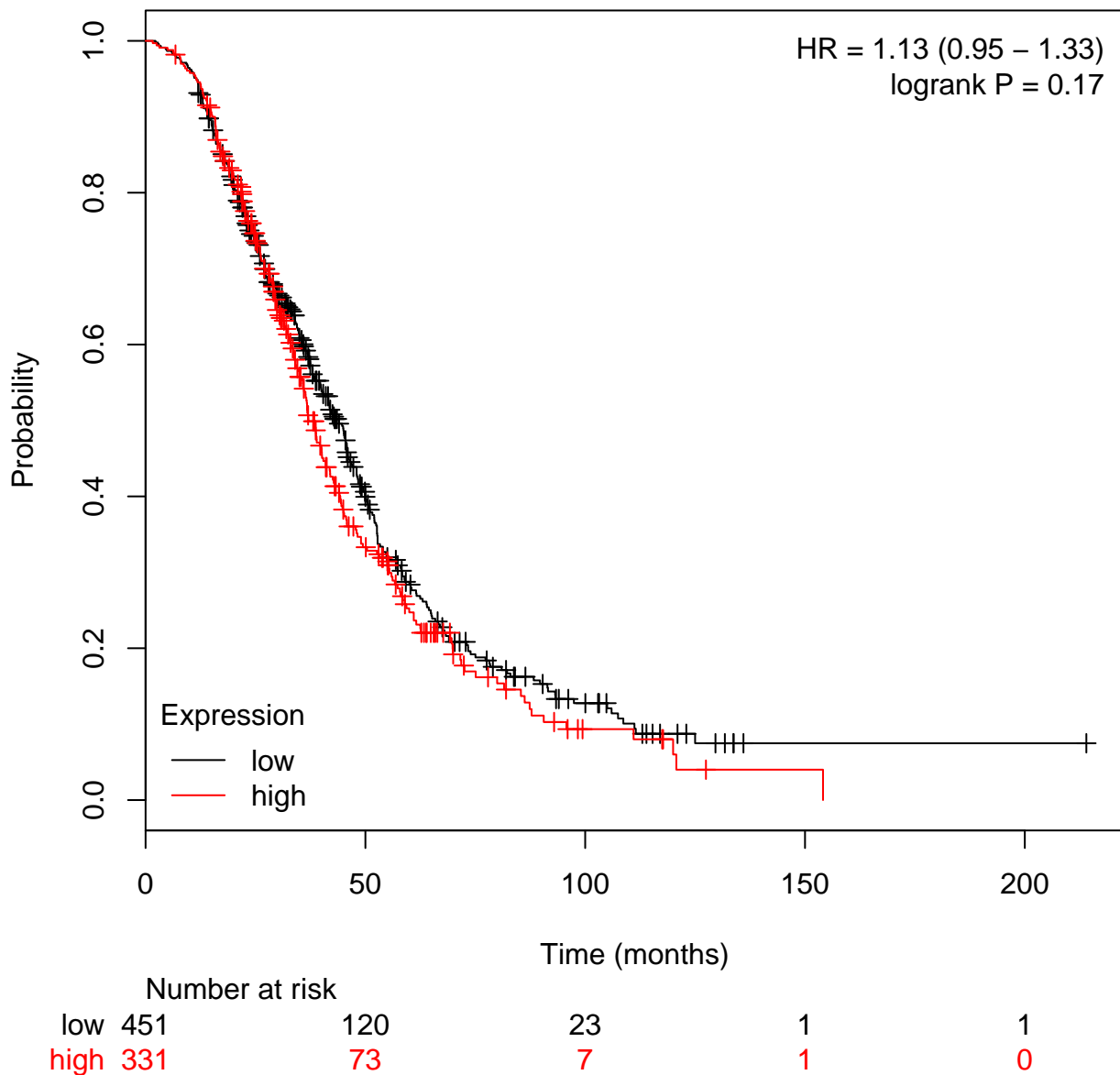

Supplement: Appendix — The prognostic value of mRNA level of E2F family members in OC patients using Kaplan-Meier plotter (p > 0.05). [file Data_Sheet_1.zip › E2F3-PPS_203692_s_at.pdf]

# 202248\_at

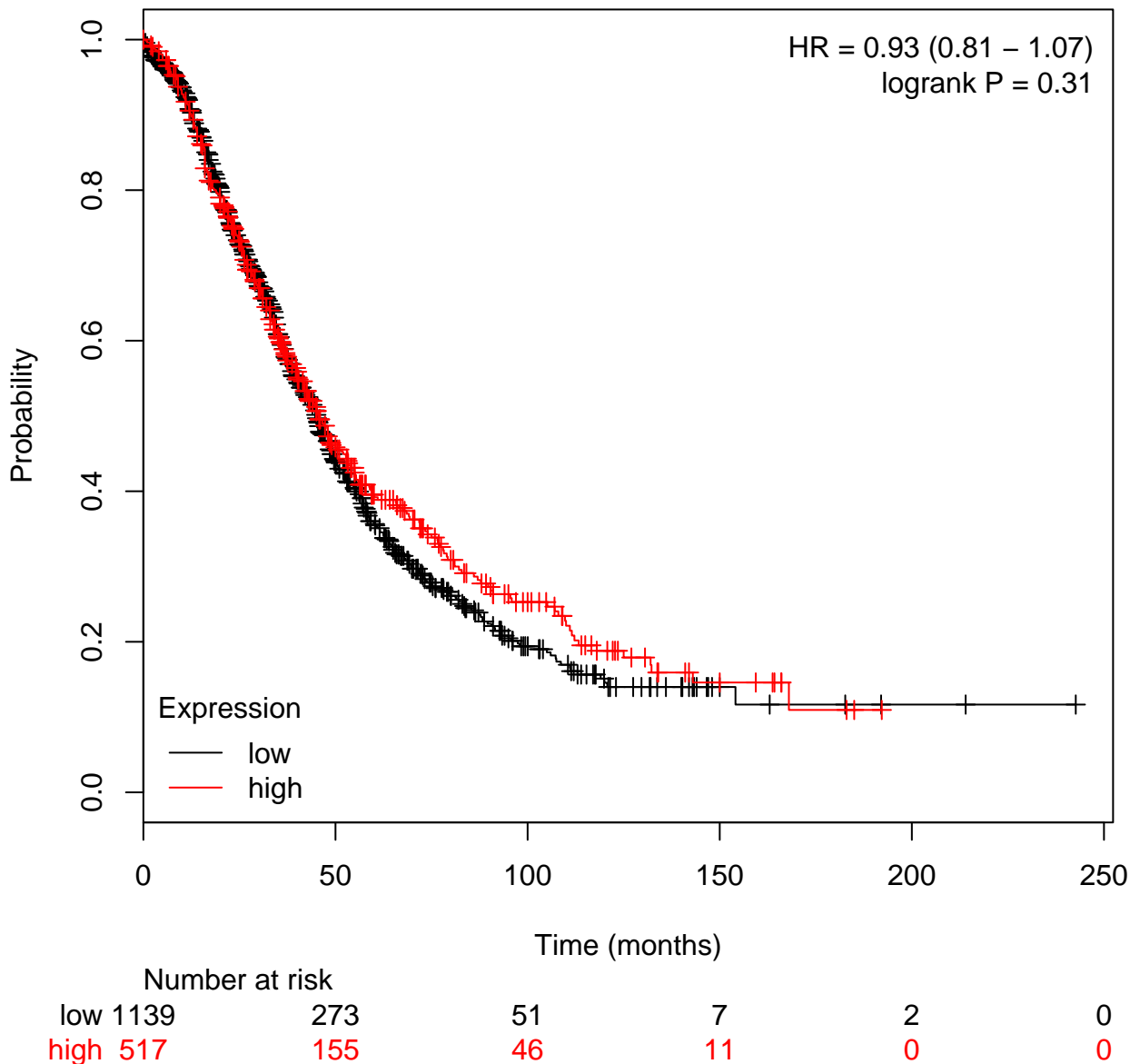

Supplement: Appendix — The prognostic value of mRNA level of E2F family members in OC patients using Kaplan-Meier plotter (p > 0.05). [file Data_Sheet_1.zip › E2F4-OS_202248_at.pdf]

# 221586\_s\_at

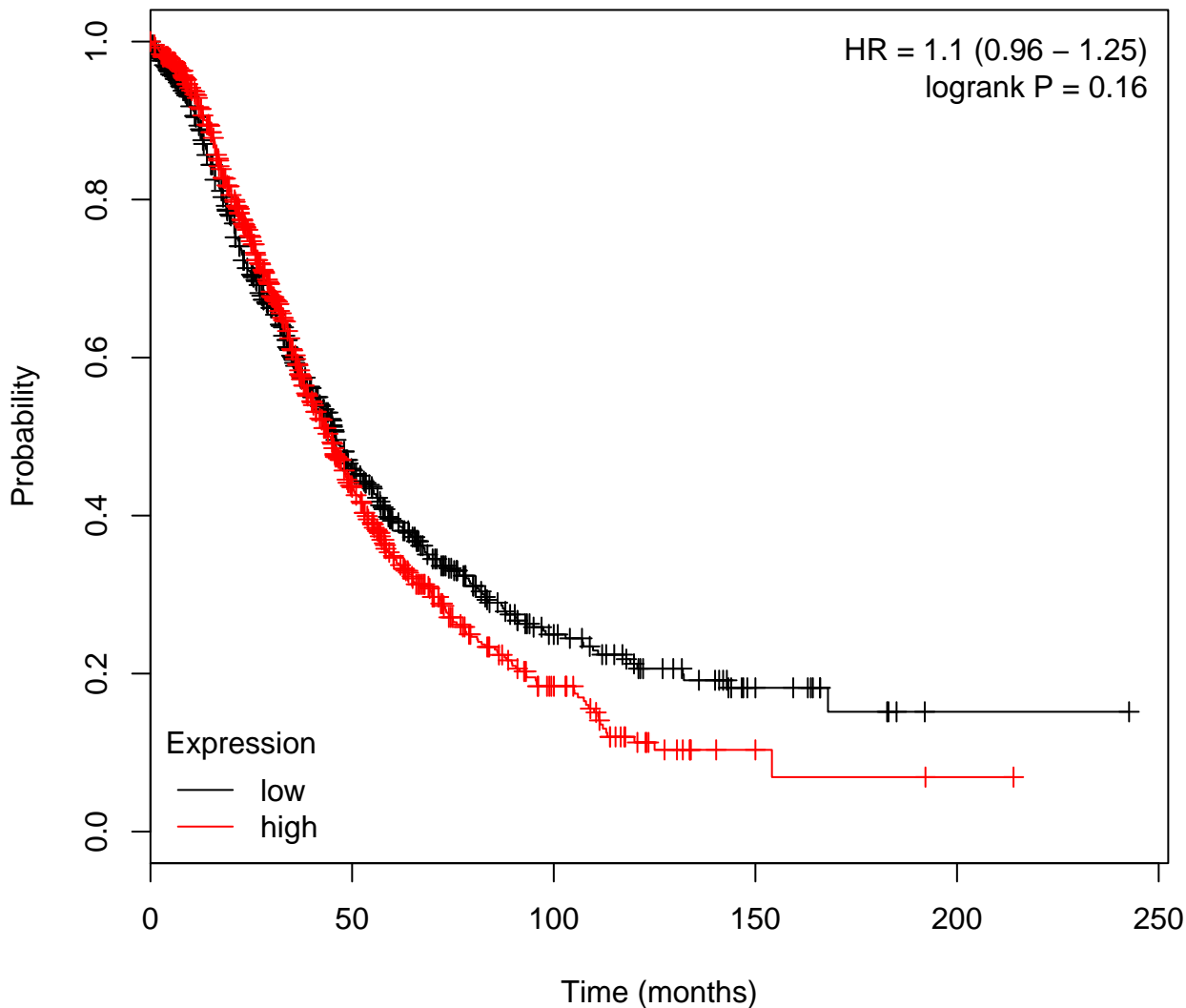

Number at risk

|      |     |     |    |    |   |   |
|------|-----|-----|----|----|---|---|
| low  | 710 | 207 | 53 | 14 | 1 | 0 |
| high | 946 | 221 | 44 | 4  | 1 | 0 |

Supplement: Appendix — The prognostic value of mRNA level of E2F family members in OC patients using Kaplan-Meier plotter (p > 0.05). [file Data_Sheet_1.zip › E2F5-OS_221586_s_at.pdf]

# 221586\_s\_at

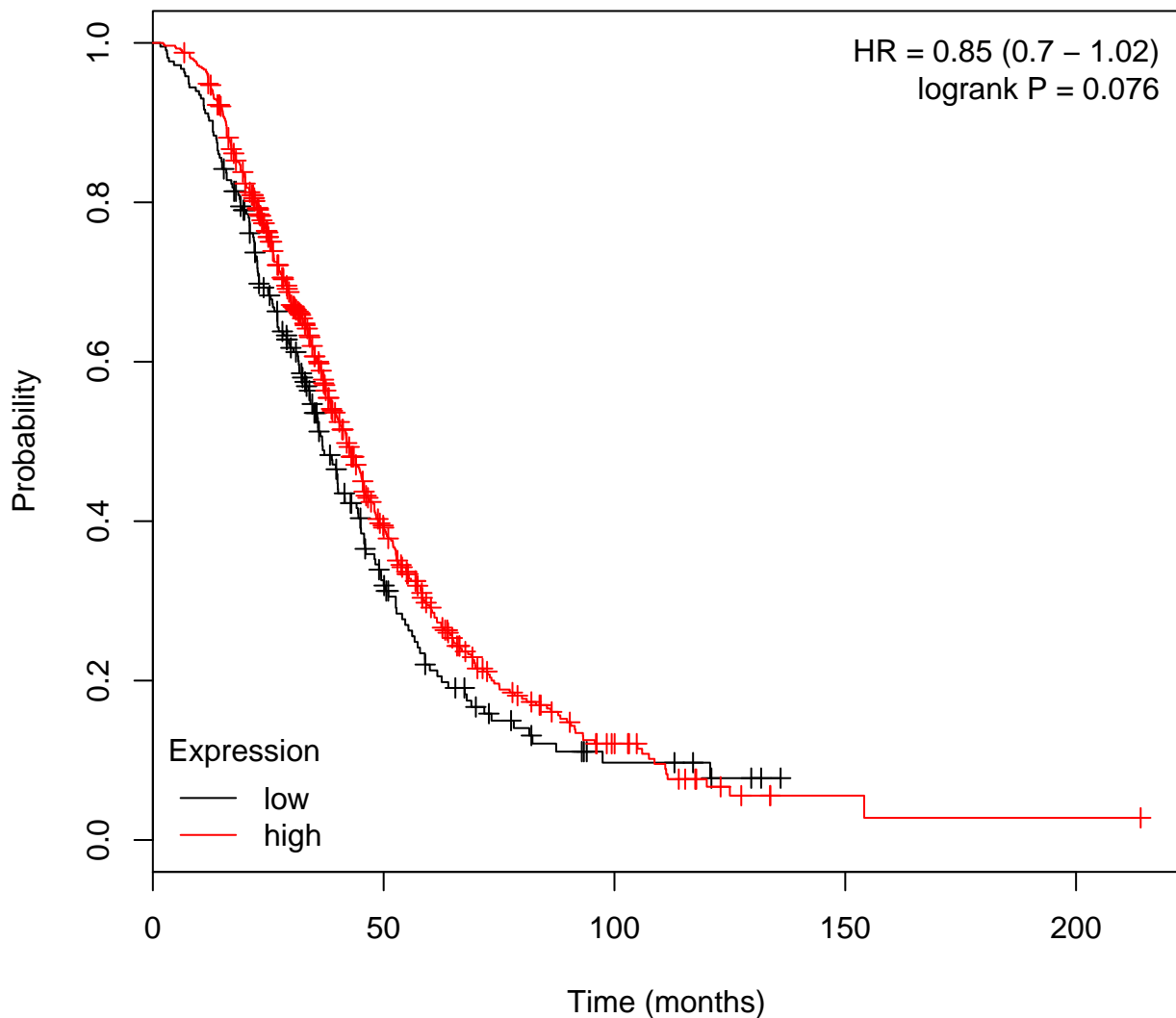

Number at risk

|      |     |     |    |   |   |
|------|-----|-----|----|---|---|
| low  | 215 | 49  | 7  | 0 | 0 |
| high | 567 | 144 | 23 | 2 | 1 |

Supplement: Appendix — The prognostic value of mRNA level of E2F family members in OC patients using Kaplan-Meier plotter (p > 0.05). [file Data_Sheet_1.zip › E2F5-PPS_221586_s_at.pdf]

# 203957\_at

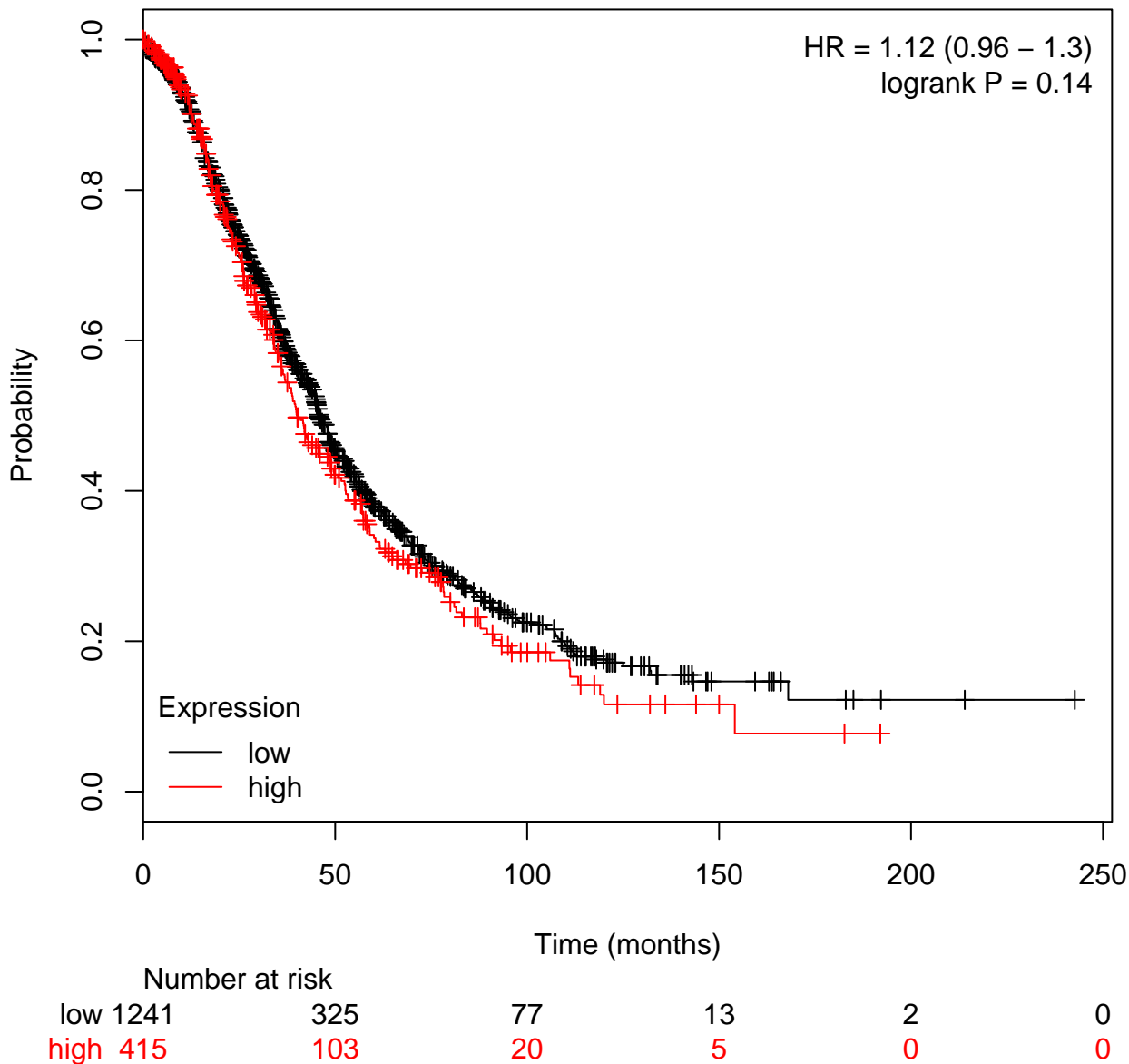

Supplement: Appendix — The prognostic value of mRNA level of E2F family members in OC patients using Kaplan-Meier plotter (p > 0.05). [file Data_Sheet_1.zip › E2F6-OS_203957_at.pdf]

## 203957\_at

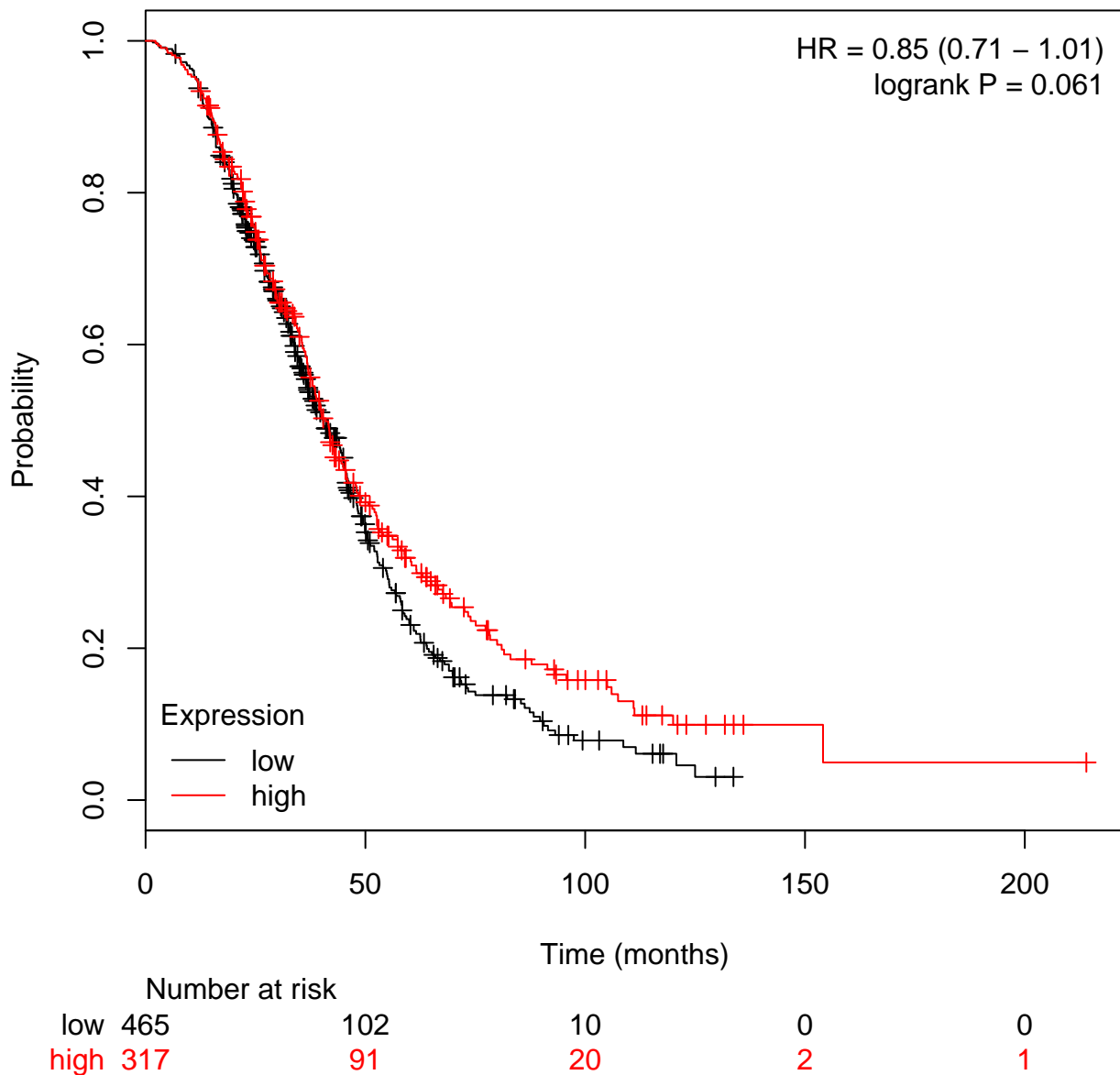

Supplement: Appendix — The prognostic value of mRNA level of E2F family members in OC patients using Kaplan-Meier plotter (p > 0.05). [file Data_Sheet_1.zip › E2F6-PPS_203957_at.pdf]

## 228033\_at

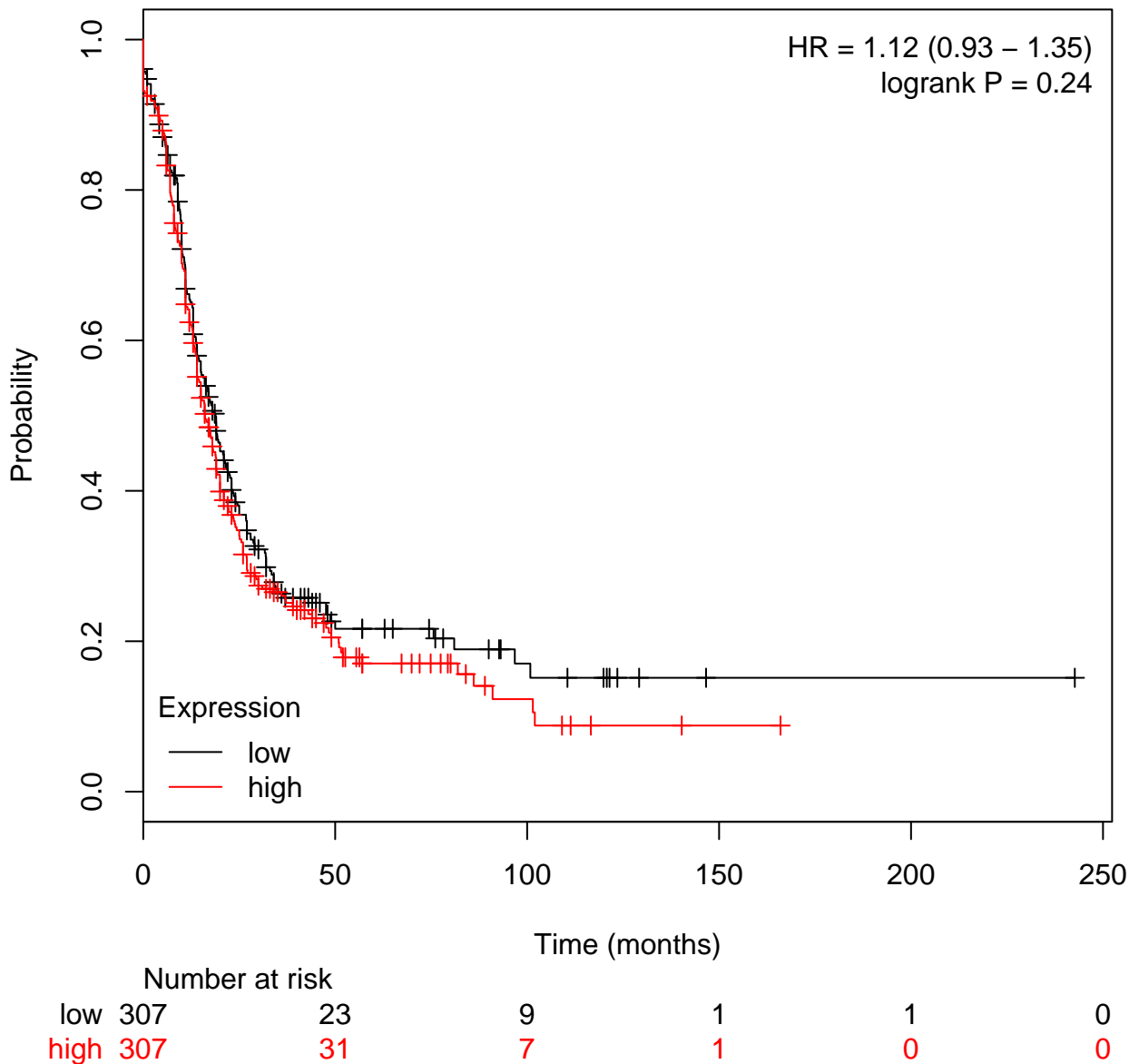

Supplement: Appendix — The prognostic value of mRNA level of E2F family members in OC patients using Kaplan-Meier plotter (p > 0.05). [file Data_Sheet_1.zip › E2F7-PFS_228033_at.pdf]

## 219990\_at

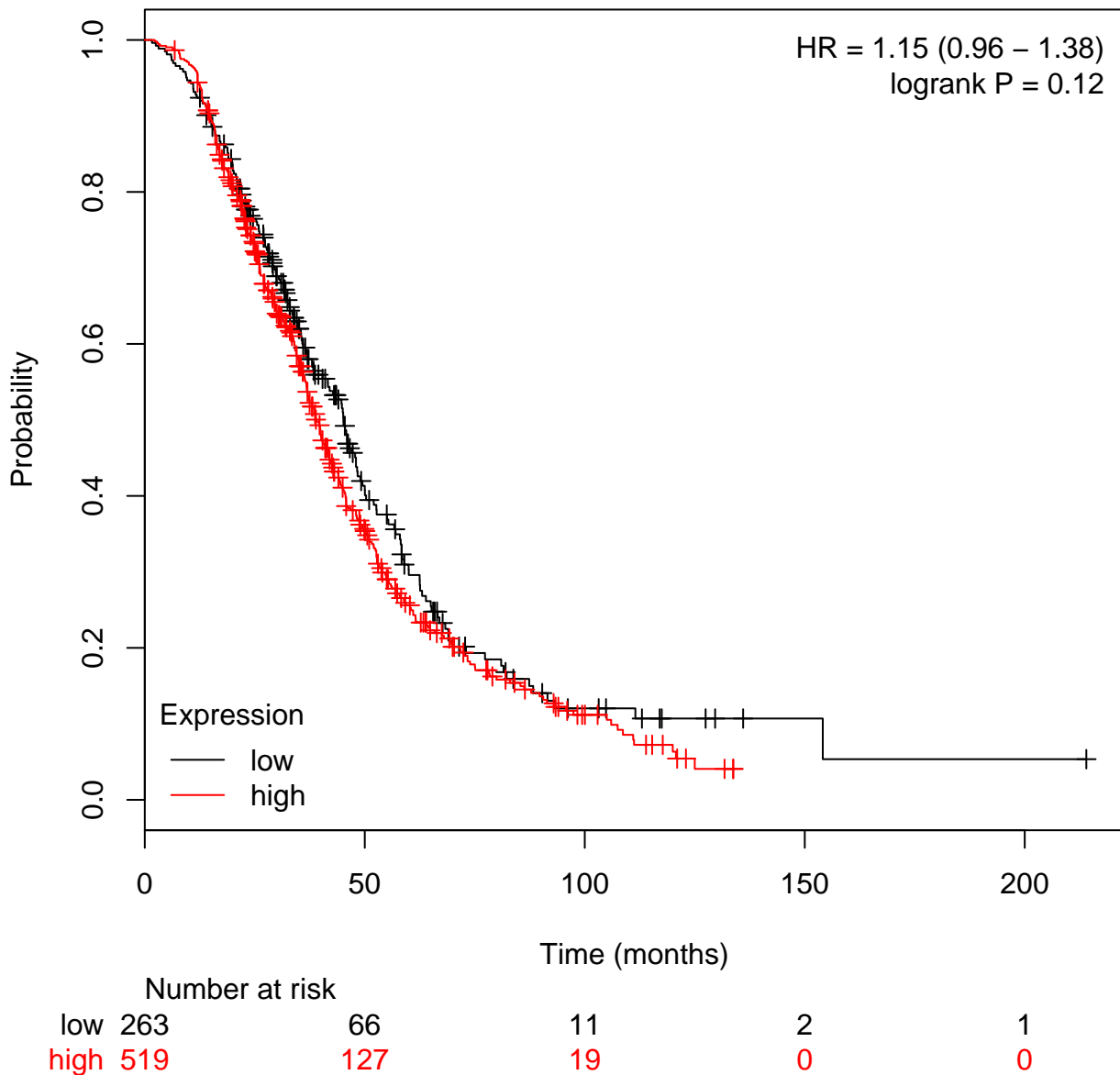

Supplement: Appendix — The prognostic value of mRNA level of E2F family members in OC patients using Kaplan-Meier plotter (p > 0.05). [file Data_Sheet_1.zip › E2F8-PPS_219990_at.pdf]
